# Supplementary material for: Comparison of seven modelling algorithms for γ‐aminobutyric acid–edited proton magnetic resonance spectroscopy
Source: NMR Biomed. 2022 Feb 23;35(7):e4702. doi: 10.1002/nbm.4702 (PMC9203918; doi:10.1002/nbm.4702)
Supplement: Supplementary file 1 — Table S1: Basic demographics, hardware and software parameters for the constituent datasets Supplementary Figure 1: Average metabolite and baseline (where applicable) models with corresponding residuals for each algorithm, baseline model and constraint model in the exploratory analysis. Corresponding fits over the full range are presented in Supplementary Figure 2. Supplementary Figure 2: Average metabolite and baseline (where applicable) models for each algorithm, baseline model and constraint model in the exploratory analysis. Supplementary Figure 3: Distribution of metabolite estimates for GABA+ (a), GABA (b), and MM3co (c) obtained from each modelling strategy, grouped by vendor Supplementary Table 2: Mean concentration estimates (institutional units, ≈ mM), for each algorithm in the exploratory analysis. Estimates grouped by vendor are expressed as a % difference relative to the mean across all subjects for the respective algorithm; significance indicated by *, **, *** for pholm < .05, .01 and .001 respectively Supplementary Figure 4: Relationship between GABA+ and grey matter, with different baseline and soft constraint parameters for the MM3 component. Robust (skipped) correlation coefficients are reported, with line‐of‐best‐fit in dashed black Supplementary Figure 5: Relationship between GABA and grey matter, with different baseline and soft constraint parameters for the MM3 component. Robust (skipped) correlation coefficients are reported, with line‐of‐best‐fit in dashed black Supplementary Table 3 Parameters for water‐scaled metabolite estimates Supplementary Table 4 Relaxation parameters used for water scaling in FSL‐MRS (all other parameters are per Supplementary Table 3) Supplementary Table 5: Relaxation parameters used for water scaling in Osprey (all other parameters per Supplementary Table 3) Supplementary Figure 6: Quality control; rejected fits for each criterion, according to algorithm. A single dataset may be flagged by multiple rejection criteri [file NBM-35-0-s001.pdf]

Supplementary Material

A Acquisition Details 3
B Exploratory Analysis: co-edited macromolecule handling and baseline interaction 4
B.1 Modelling Outcomes 6
B.2 Discussion 13
C Difference spectrum modelling 15
C.1 FSL-MRS 15
C.2 Gannet 16
C.3 jMRUI: AMARES 16
C.4 jMRUI: QUEST 17
C.5 LCModel 18
C.6 Osprey 19
C.7 Tarquin 19
D Water Reference: Modelling and Quantification 21
D.1 FSL-MRS 24
D.2 Gannet 24
D.3 jMRUI: AMARES 25
D.4 jMRUI: QUEST 25
D.5 LCModel 25
D.6 Osprey 26
D.7 Tarquin 27
E Creatine Reference: Edit-OFF sub-spectrum modelling 28
E.1 FSL-MRS 28
E.2 Gannet 29
E.3 jMRUI: AMARES 29
E.4 jMRUI: QUEST 29
E.5 LCModel 29
E.6 Osprey 30
E.7 Tarquin 30
F Quality Control 31
G Fitting Outcomes per algorithm 33
H Agreement between algorithms 35

I Additional Correlation Analyses 42
I.1 Glx_{diff}, Glx_{edit_off} 43

A Acquisition Details

Site	N	Age (years±SD)	Sex F / M	Scanner vendor and model	Software release	Tx/Rx hardware	MEGA-PRESS sequence variant	Phase cycling	Editing inter-leaving	B0 shimming approach	Water suppr.	Spectral width (Hz)	Data points
G1	7	22.9±3.7	4 / 3	GE Discovery MR750w	DV25	Body coil/32-ch head coil	Interleaved sequence	2	2	Double-echo GRE	CHESS	5000	4096
G4	12	25.6±4.5	6 / 6	GE Discovery MR750	DV25	Body coil/8-ch head coil	ATSM patch	8	1	Double-echo GRE	CHESS	5000	4096
G5	12	25.5±3.7	5 / 7	GE Discovery MR750	DV25	Body coil/32-ch head coil	ATSM patch	8	1	Double-echo GRE	CHESS	2000	2048
G6	12	24.3±4.2	6 / 6	GE Signa HDx	HD16	Body coil/8-ch head coil	ATSM patch	2	2	Double-echo GRE	CHESS	2000	2048
G7	12	28.1±4.0	6 / 6	GE Discovery MR750	DV24	Body coil/8-ch head coil	ATSM patch	8	1	Double-echo GRE	CHESS	2000	2048
G8	12	29.7±2.1	6 / 6	GE Discovery MR750	DV24	Body coil/8-ch head coil	ATSM patch	8	1	Double-echo GRE	CHESS	2000	2048
**All G**	**67**	**26.2±4.3**	**33 / 34**										
P1	9	25.0±3.7	4 / 5	Philips Achieva	R5.1.7	Body coil/32-ch head coil	JHU patch	16	1	PB-auto	VAPOR	2000	2048
P3	12	25.1±2.9	6 / 6	Philips Achieva	R3.2.2	Body coil/32-ch head coil	JHU patch	16	1	PB-auto	VAPOR	2000	2048
P4	12	29.2±3.1	5 / 7	Philips Ingenia CX	R5.1.7	Body coil/32-ch head coil	JHU patch	16	1	PB-auto	MOIST	2000	2048
P5	12	24.9±4.3	7 / 5	Philips Achieva TX	R5.1.7	Body coil/32-ch head coil	JHU patch	16	1	PB-auto	MOIST	2000	2048
P6	8	23.1±2.4	3 / 5	Philips Achieva	R3.2.3	Body coil/8-ch head coil	JHU patch	16	1	PB-auto	MOIST	2000	2048
P7	12	27.3±3.7	7 / 5	Philips Ingenia	R5.1.8	Body coil/32-ch head coil	JHU patch	16	1	PB-auto	VAPOR	2000	2048
P8	12	23.6±3.7	6 / 6	Philips Ingenia CX	R5.1.8	Body coil/32-ch head coil	JHU patch	16	1	PB-auto	MOIST	2000	2048
P9	12	23.2±2.0	5 / 7	Philips Achieva	R5.1.7	Body coil/32-ch head coil	JHU patch	16	1	PB-auto	VAPOR	2000	2048
P10	12	25.8±4.6	6 / 6	Philips Ingenia	R5.1.9	Body coil/15-ch head coil	JHU patch	16		PB-auto	MOIST	2000	2048
**All P**	**101**	**25.4±3.9**	**49 / 52**										
S1	12	25.7±3.7	6 / 6	Siemens Trio	VB17	Body coil/32-ch head coil	WIP (529)	16	1	3D-DESS + manual	CHESS	4000	4096
S3	12	31.6±3.4	9 / 3	Siemens Prisma	VD13	Body coil/20-ch head/neck coil	WIP (859D)	16	1	FAST(EST) MAP	WET	4000	4096
S5	12	26.5±3.7	6 / 6	Siemens Trio	VB17	Body coil/12-ch head coil	WIP (529)	16	1	3D-DESS	CHESS	4000	4096
S6	6	26.2±2.0	1 / 5	Siemens Trio	VB17	Body coil/32-ch head coil	WIP (529)	16	1	FAST(EST) MAP	WET	4000	4096
S8	12	24.0±3.5	11 / 1	Siemens Prisma	VE11	Body coil/64-ch head coil	WIP (859G)	16		3D-DESS	WET	4000	4096
**All S**	**54**	**26.9±4.3**	**33 / 21**										
**Total**	**222**	**26.0±4.1**	**115 / 107**										

Supplementary Table 1: Basic demographics, hardware and software parameters for the constituent datasets

B Exploratory Analysis: co-edited macromolecule handling and baseline interaction

The incompletely characterised co-edited signals underlying the GABA peak at 3 ppm present a challenge for accurate modelling of the GABA signal in the area. Whilst the main analysis considers the impact of including a simulated basis component representing that macromolecule signal (MM3co: 14 Hz FWHM gaussian centred at 3 ppm), both Osprey and LCModel have the possibility to constrain this component and to adjust baseline flexibility, potentially improving fitting outcomes further. Therefore, a series of variations were assayed as follows:

- **LCModel (no BL):** standard basis set consisting of GABA, Glu, Gln, GSH, NAA, NAAG, MM 0.9, but no MM3co; this is denoted "LCModel" in the main article.
- **LCModel (no BL) +MM3**: as above, with the addition of MM3co to the basis set; this is denoted "LCModel (+MM3)" in the main article
- **LCModel 0.6 BL**: standard basis set, no MM3co component, with a relatively stiff but non-zero cubic spline baseline model:
    - `NOBASE=F` to re-enable baseline modelling (which is disabled by default for MEGA-PRESS when using `SPTYPE=mega-press-3`),
    - `DKNTMN=0.6` to set the baseline knot spacing to 0.6 ppm (the default value is 0.15).
- **LCModel 0.6 BL +MM3**: as above, with the addition of MM3co to the basis set (no explicit constraint)
- **LCModel 0.6 BL 1:1 GABA**: as above, with the addition of a 1:1 soft constraint between GABA and MM3co
    - `NRATIO=2` Two soft constraints will be specified (see below…)
    - `CHRATO(1)='NAAG/NAA = 0.14 +- 0.15'` represents LCModel's default soft constraint for NAAG/NAA. LCModel defines several other default constraints,

not applied due to their dependence on components not included in the standard basis set.

- `CHRATO(2)='GABA/MM3co = 1.0 +- 0.1'` applies a soft constraint on the ratio of GABA to MM3co, with an expected value of 1.0 and standard deviation of 0.1.

- **LCModel 0.6 BL 3:2 MM09**: as above, but with a 3:2 soft constraint between MM3co and MM0.9 (and no constraint between MM3co and GABA), as proposed in [1]:
  - `CHRATO(2)='MM3co/MM09ex = 0.66 +- 0.2'`
- **Osprey 0.4 BL**: standard basis set, without the simulated MM3co component. Default 0.4ppm baseline knot spacing. Denoted "Osprey" in the main article.
- **Osprey 0.6 BL**: as above, but with broader 0.6ppm baseline knot spacing
  - `bLineKnotSpace = 0.6`
- **Osprey 0.4 BL +MM3**: same as Osprey 0.4 BL, with the MM3co component included but no constraint applied:
  - `coMM3 = 'freeGauss14'`
- **Osprey 0.4 BL 1:1 GABA**: as with Osprey 0.4 BL +MM3, with a 1:1 soft constraint between MM3co and GABA amplitudes:
  - `coMM3 = '1to1GABAsoft'`
- **Osprey 0.6 BL 1:1 GABA**: as above, with broader 0.6ppm baseline knot spacing – a comparable configuration to the LCModel 0.6 BL 1:1 GABA case.
- **Osprey 0.4 BL 3:2 MM0.9**: as with Osprey 0.4 BL +MM3, but with a 3:2 soft constraint between the simulated MM3co component and MM0.9 amplitude
  - `coMM3 = '3to2MMsoft'`
- **Osprey 0.6 BL 3:2 MM0.9**: as above, with broader 0.6ppm baseline knot spacing – comparable with LCModel 0.6 BL 3:2 MM09

B.1 Modelling Outcomes

On visual inspection (refer Supplementary Figure 1), a broader baseline knot spacing for Osprey yields seemingly more complete coverage of the apparent GABA signal when modelled without an explicit MM3 component. However, the stiffer baseline model for Osprey showed a dip in the 3.0 ppm region when the MM3co component was included, suggesting a possible over-estimation of GABA+ area. Inclusion of MM3 in the model resulted in substantially lower residual signal visible around 3.0 ppm for both Osprey and LCModel. Residuals from LCModel modelling showed few discernible differences between the different soft constraint strategies, while the 3:2 constraint to MM 0.9 for Osprey yielded slightly reduced residuals compared to the 1:1 GABA constraint model around 3.0 – 3.2 ppm.

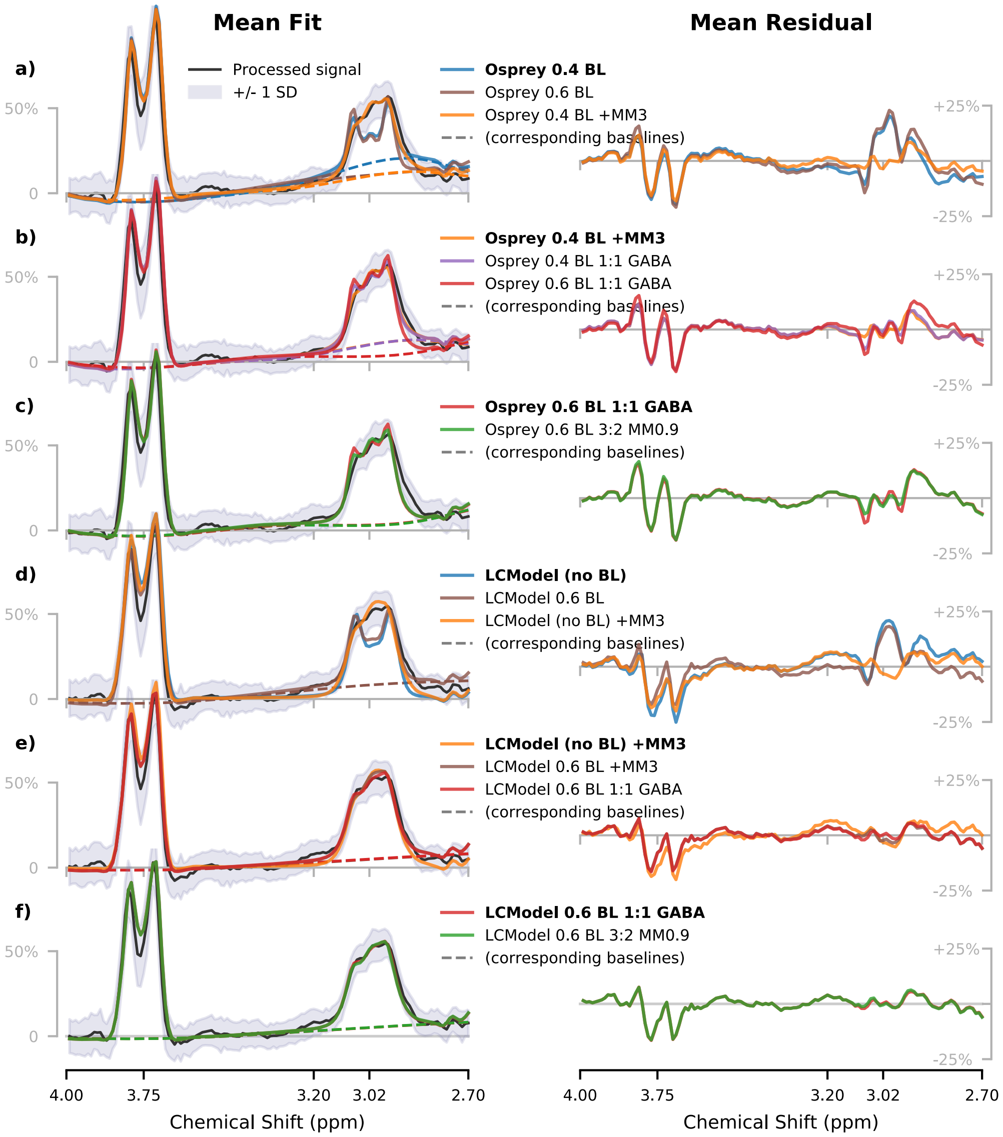

Supplementary Figure 1: Average metabolite and baseline (where applicable) models with corresponding residuals for each algorithm, baseline model and constraint model in the exploratory analysis. Corresponding fits over the full range are presented in Supplementary Figure 2.

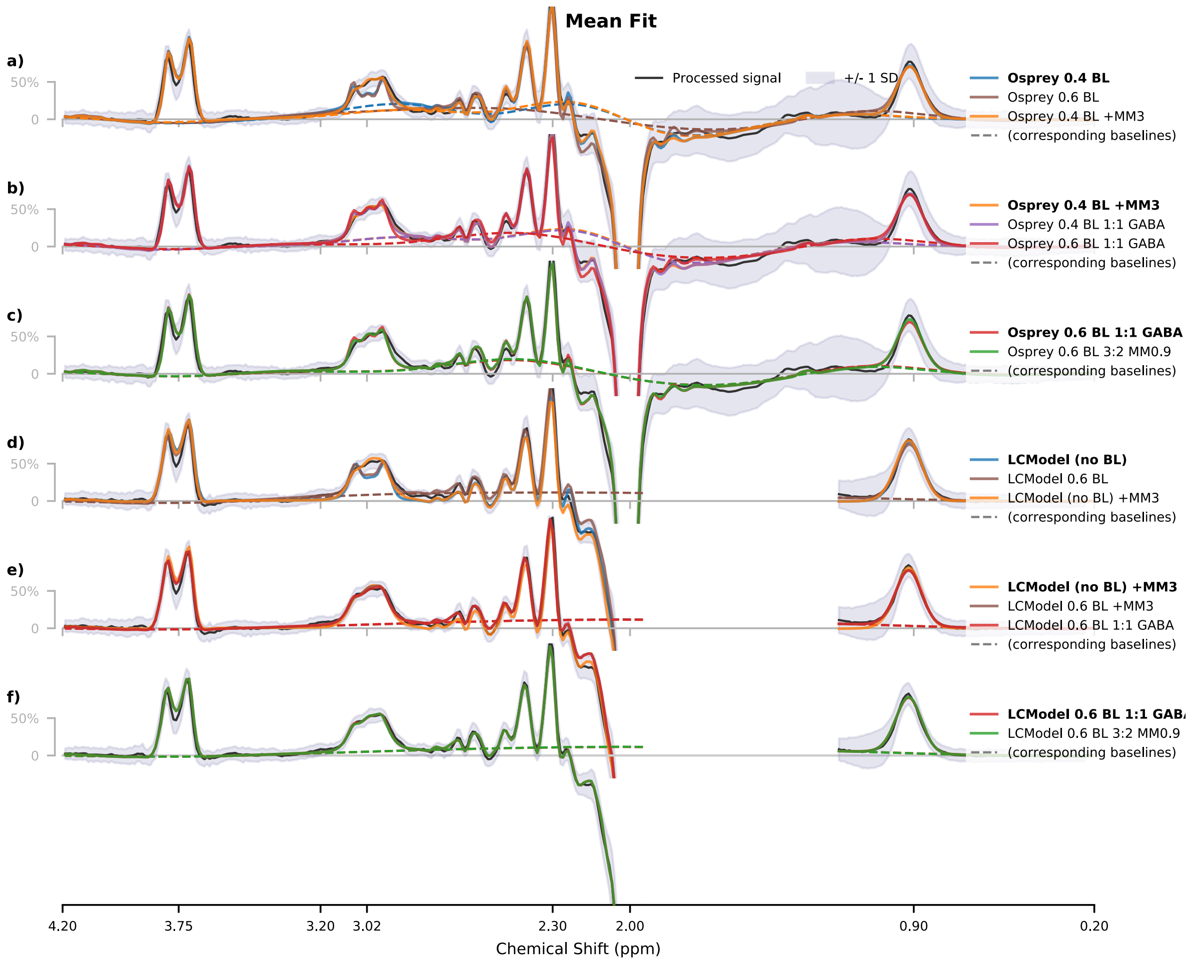

Supplementary Figure 2: Average metabolite and baseline (where applicable) models for each algorithm, baseline model and constraint model in the exploratory analysis.

Groupwise statistics are summarised in Supplementary Figure 3 and reported in Supplementary Table 2. Inclusion of the MM3co component appeared to mitigate vendor-specific effects for GABA+/H_2O for both algorithms (p_{holm} > 0.05 for most configurations). However, strong, complementary vendor differences were seen for both separate components, GABA/H_2O and MM3co/H_2O. For Siemens datasets, GABA/H_2O estimates were systematically lower at trend level (median across configurations -12.6%, n.s.), while MM3co/H_2O estimates were elevated (median across configurations +15.3%, p_{holm} < 0.01).

Correlation between GABA+/H_2O estimates and grey matter fraction (Supplementary Figure 4) was comparable across all configurations. While the results suggest somewhat better performance for LCModel 0.6 BL (regardless of the constraint model for MM3), this difference did not approach statistical significance (p_{holm}>>0.05).

For separated GABA/H_2O (Supplementary Figure 5), the LCModel 0.6 BL configuration (having no explicit MM3 component in the model) showed the highest correlation with tissue fraction, although differences between algorithms were not found to be statistically significant. Relative to the GABA+/H_2O estimates from each configuration, only the LCModel 0.6 BL 1:1 GABA configuration (with a 1:1 soft constraint between MM3 and GABA amplitudes) showed significantly degraded correlation ($p_{holm}<0.05$). Differences for Osprey were subtle and non-significant.

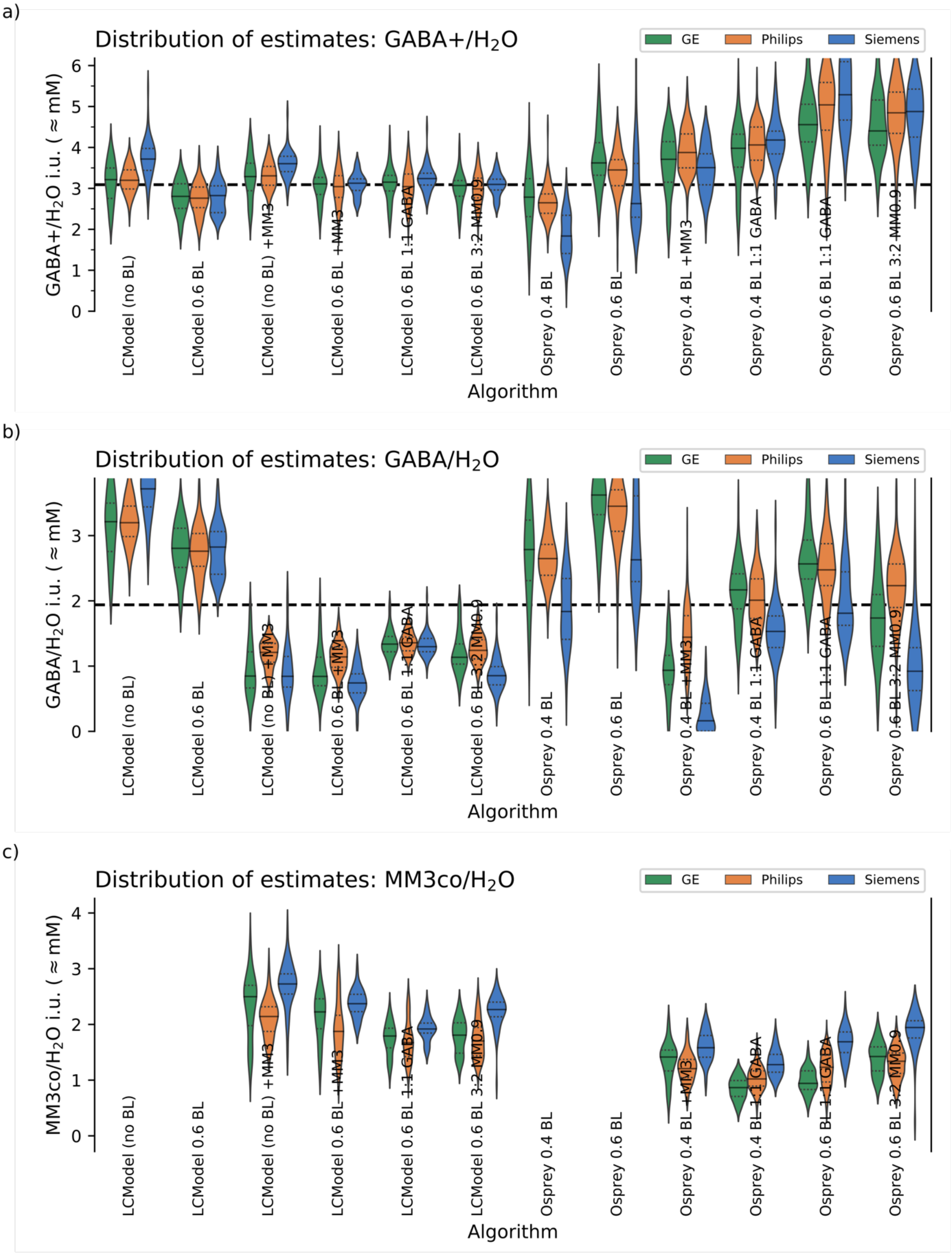

Supplementary Figure 3: Distribution of metabolite estimates for GABA+ (a), GABA (b), and MM3co (c) obtained from each modelling strategy, grouped by vendor

	LCModel (no BL)	LCModel 0.6 BL	LCModel (no BL) +MM3	LCModel 0.6 BL +MM3	LCModel 0.6 BL 1:1 GABA	LCModel 0.6 BL 3:2 MM0.9	Osprey 0.4 BL	Osprey 0.6 BL	Osprey 0.4 BL +MM3	Osprey 0.4 BL 1:1 GABA	Osprey 0.6 BL 1:1 GABA	Osprey 0.6 BL 3:2 MM0.9	Median
GABA+/H2O mean ±SD	3.302 ±0.484	2.777 ±0.387	3.378 ±0.408	3.100 ±0.388	3.139 ±0.406	3.045 ±0.362	2.536 ±0.640	3.451 ±0.741	3.710 ±0.619	4.064 ±0.608	4.925 ±0.912	4.763 ±0.784	3.191 ±0.479
GABA+/H2O diff GE	-2.7%	+1.0%	-2.6%	+0.3%	+0.4%	+0.8%	+9.9%	+4.9%	+0.0%	-2.1%	-7.5%	-7.6%	-0.4%
GABA+/H2O diff Philips	-3.2%	-0.6%	-2.1%	-1.8%	-3.1%	-1.9%	+4.4%	-0.0%	+4.5%	-0.1%	+2.3%	+1.7%	-2.0%
GABA+/H2O diff Siemens	*** +12.5%	+1.7%	** +6.7%	+0.9%	+3.2%	+1.7%	*** -27.6%	-23.8%	-5.5%	+2.9%	+7.4%	+2.4%	+5.2%
GABA/H2O mean ±SD	3.302 ±0.484	2.777 ±0.387	1.088 ±0.348	0.976 ±0.316	1.338 ±0.176	1.143 ±0.296	2.536 ±0.640	3.451 ±0.741	1.026 ±0.635	1.912 ±0.489	2.451 ±0.570	1.891 ±0.802	1.896 ±0.410
GABA/H2O diff GE	-2.7%	+1.0%	-22.1%	-13.7%	-0.1%	-0.7%	+9.9%	+4.9%	-8.8%	+13.4%	+4.7%	-8.1%	+8.6%
GABA/H2O diff Philips	-3.2%	-0.6%	** +11.2%	*** +16.9%	+1.4%	* +8.6%	+4.4%	-0.0%	*** +40.6%	+5.1%	+1.0%	*** +18.1%	+1.5%
GABA/H2O diff Siemens	*** +12.5%	+1.7%	-22.5%	*** -24.0%	-3.0%	*** -25.3%	*** -27.6%	-23.8%	*** -84.2%	*** -19.9%	** -26.1%	*** -51.3%	-12.6%
MM3co/H2O mean ±SD			2.321 ±0.484	2.117 ±0.465	1.769 ±0.322	1.855 ±0.378			1.358 ±0.320	1.031 ±0.269	1.225 ±0.366	1.444 ±0.379	1.746 ±0.411
MM3co/H2O diff GE			+7.6%	+5.1%	+1.3%	-2.6%			+4.2%	*** -16.0%	*** -23.1%	-1.4%	-0.6%
MM3co/H2O diff Philips			** -7.7%	-11.5%	-5.5%	-8.2%			* -11.2%	-0.9%	+0.5%	** -7.2%	-6.4%
MM3co/H2O diff Siemens			*** +17.5%	*** +12.0%	** +8.4%	*** +22.2%			*** +16.5%	*** +23.9%	*** +37.9%	*** +34.7%	** +15.3%

*Supplementary Table 2: Mean concentration estimates (institutional units, ≈mM), for each algorithm in the exploratory analysis. Estimates grouped by vendor are expressed as a % difference relative to the mean across all subjects for the respective algorithm; significance indicated by *, **, *** for p_{holm} < .05, .01 and .001 respectively*

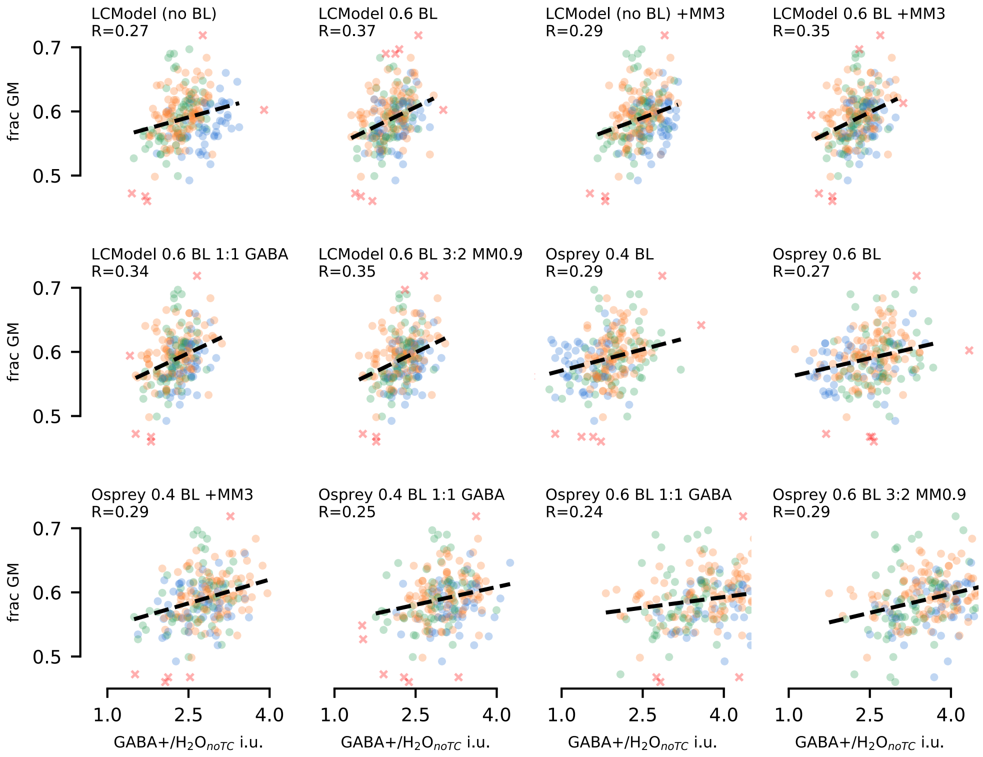

Supplementary Figure 4: Relationship between GABA+ and grey matter, with different baseline and soft constraint parameters for the MM3 component. Robust (skipped) correlation coefficients are reported, with line-of-best-fit in dashed black

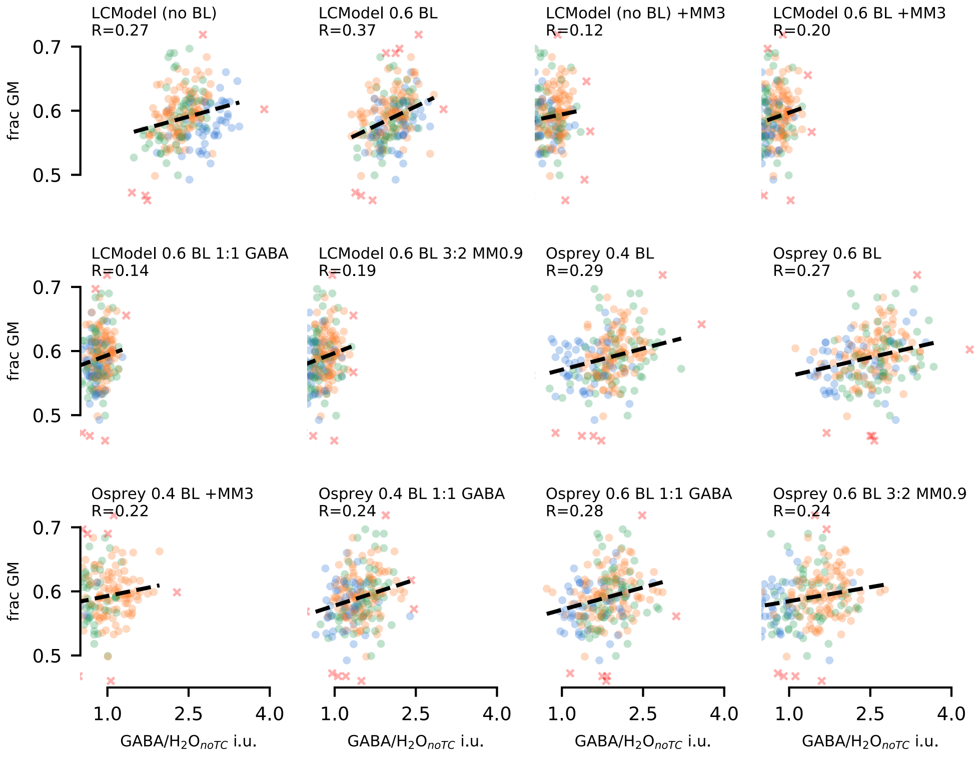

Supplementary Figure 5: Relationship between GABA and grey matter, with different baseline and soft constraint parameters for the MM3 component. Robust (skipped) correlation coefficients are reported, with line-of-best-fit in dashed black

B.2 Discussion

Whilst a flexible baseline can provide a visually appealing model, i.e., reduce apparent residuals, this does not necessarily lead to more accurate metabolite estimates. This is particularly apparent for the case of Osprey (without the MM3co component), where residuals associated with macromolecule contamination and the 3.2 ppm artefact pull the baseline far into the peak at 3 ppm, potentially leading to a possible under-estimation of the corresponding GABA+ signal. In the present study, stiffer baseline models appeared to be more robust towards small artefacts, allowing the model to reasonably follow the Gaussian contour of the edited GABA+ signal without absorbing too much of it into the baseline.

Nonetheless, using correlation with grey matter fraction as a benchmark, the performance of each algorithm without an explicit MM3 basis component, allowing a stiff but non-zero baseline to absorb the macromolecule signal, was comparable with more elaborate constrained configurations for assessment of GABA+. Moreover, when reporting GABA separately, such a baseline model showed a tendency towards better performance (for LCModel) than other approaches. All configurations except LCModel 0.6 BL 1:1 GABA

were able to assess GABA/H_2O separately with comparable effectiveness to GABA+/H_2O from the same algorithm. However, we note that elevated MM3 content in white matter [2,3] may moderate correlation in the case of GABA+. Divergent outcomes for separate GABA/H_2O and MM3co/H_2O estimates across different vendors may be of concern, and requires further attention.

C Difference spectrum modelling

Further details on specific parameters adopted for modelling the difference spectrum by each algorithm are presented here; this supplements details in the main article, section 2.3.

Basis set construction is described in the main paper, section 2.3.1. Basis sets were exported both in their original resolution (8192 samples at 4 kHz sweep width) and after resampling to match the resolution of the acquired data for each manufacturer/site, to cater for those algorithms which were unable to resample effectively internally (QUEST, Tarquin). The LCModel basis set format was used as an intermediary for import into LCModel, Tarquin and FSL-MRS, while individual jMRUI-format text files were required for assembly into a jMRUI-compatible metabolite list for QUEST.

C.1 FSL-MRS

FSL-MRS [4] models data as a linear combination of basis components in the frequency domain, using Bayesian statistics for optimization. Version 1.1.1 was used, which includes updates to the calculation of water-scaled estimates. The standard basis sets as described in section 2.3.1 are used, with the recommended Metropolis-Hastings algorithm and the default fit range (0.2-4.2 ppm).

A single set of shift and line-broadening parameters (for Lorentzian and Gaussian components of a Voigt lineshape model) was applied to all metabolites in the basis set: no additional shift groups were defined.

Default baseline model is a second-order polynomial fit over the defined fit range. To allow FSL-MRS to yield water-scaled estimates, NAA+NAAG was defined as an internal reference for basis set scaling, and fixed tissue content (50% white/50% grey matter, 0% CSF) were supplied. These factors were later reversed (see sections 2.4.1 and D.1).

Typical invocation of FSL-MRS for GABA-edited difference spectra was with the following parameters:

```
--basis ge_diff_8192_4000.basis
--data S12_GABA_68.diff.nii.gz
--h2o S12_GABA_68.ref.nii.gz
```

```
--output S12_GABA_68.diff
--overwrite
--algo MH
--TE 68
--TR 2.0
--tissue_frac .5 .5 0
--internal_ref NAA NAAG
--verbose
--report
--combine NAA NAAG
--combine Glu Gln GSH
--combine GABA MM3co

Defaults:
  --ppmlim:           (0.2, 4.2)
  --baseline_order:  2
  --metab_groups:    0
  --h2o_scale:       1.0
```

C.2 Gannet

Gannet [5] models data by fitting peaks in the frequency domain. Version 3.1 was used, with the 'GABA+Glx' model applied to the difference spectrum between 2.79 and 4.1 ppm. This model fits the GABA+ peak with a single Gaussian peak around 3.02 ± 0.05 ppm and Glx as a pair of Gaussian peaks at 3.71 ± 0.02 ppm and 3.79 ± 0.02 ppm, and includes terms to characterize the baseline. The Gannet baseline is modelled as a linear slope with sinusoid and cosine terms, periodic at 2.62 ppm (i.e., $A * (f - f_0) + B * \sin(\pi * f / 1.31 / 4) + C * \cos(\pi * f / 1.31 / 4)$ where f is the frequency (in ppm) and f_0 the offset to the first modelled Glx peak (±3.71 ppm).

GannetFit was run on data from the standardised processing pipeline (reported as "Gannet"), and on data processed with Gannet's own default pipeline, denoted "Gannet (native)". The latter applies zero-filling (to 0.062 Hz spectral resolution), and line-broadening (3 Hz), neither of which are performed in the standardised processing pipeline.

C.3 jMRUI: AMARES

jMRUI [6,7] is a Java-based package for time domain analysis of MRS and MRSI data, implementing several modelling algorithms. For the present study, jMRUI v6.0 beta was

used. Due to compatibility issues with contemporary operating systems, all jMRUI operations were performed on a dedicated 32-bit Debian Linux virtual machine.

AMARES [8] was configured with a GABA+Glx model equivalent to that of Gannet for fitting of the difference spectra, with additional components for NAA and the Glx C4 spins. This incorporated a simple Gaussian model for GABA+ (3.02 ± 0.05 ppm), and a dual-gaussian model for Glx (3.71 ± 0.02 ppm, 3.79 ± 0.02 ppm) – note the close similarity to the Gannet model. The model also used Lorentzian signals to fit the negative NAA (2.0 ± 0.02 ppm) and the Glx C4 spins (2.29 ± 0.02ppm, 2.39 ± 0.02 ppm). Soft constraints were applied on linewidth (5-15 Hz for all Glx, 5-25 Hz for GABA, 5-20 Hz for NAA) and relative phase (±10 degrees, offset by 180 degrees in the case of NAA). No constraints were applied between individual components. The Glx C4 components were included for completeness of modelling, but did not contribute to the reported Glx estimates. Since input data had already been phase corrected, global zero-th and first-order phase were set to zero. Baseline modelling was not performed.

C.4 jMRUI: QUEST

QUEST [9,10] is a time domain linear-combination modelling algorithm, implemented within jMRUI. Standard basis set components for each manufacturer and spectral resolution, as described in section 2.3.1, were imported in jMRUI text format and manually aligned for NAA @ 2.0 ppm, before assembly into a jMRUI-format metabolite list.

In earlier testing with the supplied NMRScope-B tool and the supplied megapress_2edit_voi_1pws protocol (configured for TE = 68 ms, TE1 = 13.1 ms, water suppression at 4.7 ppm and 15 ms, 180º gaussian editing pulses at 1.9 and 7.5 ppm adjusted to an “observation offset” of 2 ppm), both the NAAG and GSH components consistently failed to simulate: a complete basis set could not be produced with this tool alone.

The assembled metabolite lists were subsequently used to model batches of data grouped by manufacturer and spectral resolution. The QUEST algorithm was invoked with the zero-order phase fixed to 0 degrees. Other parameters were according to default: no first-order phase; damping factor α (proportional to linewidth, ΔFWHM = α/π) and

frequency shift, per basis function, set on the range -10 to 40 Hz and -10 to 10 Hz, respectively; no baseline terms were included.

C.5 LCModel

LCModel [11] models data with a linear combination of basis sets. Version 6.3-1P was used, with the standard basis set described in section 2.3.1. An initial fit was performed according to recommendations in the LCModel manual, with the “special type” set for MEGA-PRESS, on the range 0.2-4.2 ppm with a gap from 1.2-1.95 ppm; water scaling enabled, ECC disabled. This mode of operation assumes a completely flat baseline. Outcomes for this case are labelled as “LCModel”. For comparability, the analysis including a basis component representing macromolecule contributions around 3 ppm (“LCModel (+MM3)”) leaves all other factors unchanged, including the flat baseline assumption. Additional analyses incorporating a stiff but non-zero baseline and soft constraint models on the MM3 component are considered in a supplementary analysis, section B.

LCModel was invoked with the following parameters for modelling GABA-edited difference spectra:

- `SPTYPE=’mega-press-3’` sets the “special type” appropriately for MEGA-PRESS. This implies a flat baseline (`NOBASE=T`), the use of NAA @ 2.01 ppm as a reference for basis set scaling (`wsmet=’NAA’, wsppm=2.01`), and default correction factor for attenuation of NMR-visible water, ATTH2O = 0.43 (suitable for TE = 68 ms).
- `PPMST=4.2, PPMEND=0.2` model on the range 0.2-0.4 ppm…
- `PPMGAP(1,1)=1.95, PPMGAP(2,1)=1.2` …but exclude the 1.2-1.95 ppm range from modelling
- `DOWS=T` do water scaling
- `DOECC=F` disable ECC

C.6 Osprey

Osprey [12] implements a frequency domain basis set fit; version 1.0.1.1 was used, with default fit and reconstruction settings: separate fit on the range 0.2-4.2 ppm, incorporating macromolecule and lipid components in the basis set but without adding a co-edited MM3 peak. Knot spacing for the spline baseline was set at 0.4 ppm. Outcomes according to these settings are labelled "Osprey". The +MM3 variation (Osprey (+MM3)) includes the MM3co basis set component without additional constraints on amplitude. Further variations with respect to baseline knot spacing and soft constraints on MM3 amplitude are considered in a supplementary analysis, section B.

C.7 Tarquin

Tarquin [13,14] is a basis set approach which extends the QUEST algorithm for estimation of amplitudes in the time domain. A local build of Tarquin v4.3.11 was used, with the standard basis set per section 2.3.1.

Processed data and basis sets were supplied to Tarquin in the corresponding LCModel file formats; a minor bugfix (subsequently published upstream) was required for LCM basis set import (https://github.com/martin3141/tarquin/commit/cdc44df).

NAA was used as a reference for basis set scaling. Appropriate TE, sweep width and centre frequency (`echo`, `fs`, `ft`) were specified on the command line. Automatic phasing and referencing (`auto_phase` and `auto_ref`) were disabled. The start point for analysis of the difference spectra (`start_pnt`) was set at 5 ms (calculated as 0.005 times the sample frequency). Eddy-current correction was disabled (`--water_eddy false`). HSVD was performed (per defaults) to remove residual water. The visual baseline (Figure 2f) is modelled from the residuals, smoothed with a cosine kernel. Outcomes from this quantification are reported as "Tarquin".

Tarquin can internally simulate basis sets on demand, using an implementation of the density matrix formulation of NMR [15,16] and the parameters of [17]; an additional analysis was performed using this internally generated basis set: `--int_basis megapress_gaba` for the difference spectra. This is simulated assuming a simplified system of uncoupled spins, similar to that used by peak-integration algorithms, comprising GABA_A (3.04 ppm), GABA_B (2.95

ppm), GLX_A (2.299 ppm), GLX_B (2.400 ppm), GLX_C (3.707 ppm), GLX_D (3.789 ppm), NAA (2.0 ppm). Outcomes for this fit are reported as "Tarquin (internal)".

Typical parameters for tarquin, modelling GABA-edited difference spectra:

```
tarquin
--format lcm
--input S12_GABA_68.diff.RAW
--input_w S12_GABA_68.diff.REF

--echo 0.068
--fs 5000
--ft 127714400
--start_pnt 25
--pul_seq mega_press

--auto_phase false
--auto_ref false
--water_eddy false

--w_att 0.76
--w_conc 35880

--ext_pdf true
--output_txt S12_GABA_68.diff.tarquin.txt
--output_csv S12_GABA_68.diff.tarquin.csv
--output_pdf S12_GABA_68.diff.pdf
--output_fit S12_GABA_68.tarquin.fit
```

For "Tarquin" (with standard basis set, with or without MM3):

```
--basis_lcm ge_diff_4096_5000.basis
```

Or for "Tarquin (internal)":

```
--int_basis megapress_gaba
```

D Water Reference: Modelling and Quantification

While all algorithms yielded estimates scaled to water, specifics of the adjustments and correction factors available varied considerably between algorithms; correction according to tissue class was only natively available in a few algorithms (Gannet, Osprey, FSL-MRS). To ensure a fair comparison, scaling as documented for the respective algorithms was first reversed to yield a raw ratio of metabolite area over water area, adjusted by integer scaling factors to account for differing conventions during sub-spectral combination. Subsequently, water-scaled estimates in pseudo-absolute molar units, accounting for differing properties of water in each segmented tissue class, were evaluated according to equation 14 of [18]. Note that additional terms proposed in that paper to account for different metabolite signal relaxation rates in GM and WM were not included, due to currently limited literature on per-compartment relaxation properties of GABA. Constant scaling terms for assumed editing efficiency and macromolecule contamination were applied, per Equation 1. Water-scaled, tissue-class corrected molar concentration estimates are hereafter denoted "/H_2O".

$$[\mathrm{M}]_{/\mathrm{H_2O}} = \frac{S_\mathrm{M}}{S_{\mathrm{H_2O}}} \cdot [\mathrm{H_2O}] \cdot \frac{\#H_{\mathrm{H_2O}}}{\#H_{\mathrm{Met}}} \cdot \frac{\sum_{TC}^{GM,WM,CSF} f_{TC,vol} \cdot \beta_{TC} \cdot \mathrm{R}_{\mathrm{H_2O,TC}}}{\left(1 - f_{CSF,vol}\right) \cdot \mathrm{R}_{\mathrm{Met}}} \cdot \frac{MM}{\kappa}$$

Equation 1

Where

$$\mathrm{R}_{\mathrm{H_2O,TC}} = \left[1 - \exp\left(\frac{-\mathrm{TR}}{\mathrm{T1}_{\mathrm{H_2O,TC}}}\right)\right] \cdot \exp\left(\frac{-\mathrm{TE}}{\mathrm{T2}_{\mathrm{H_2O,TC}}}\right)$$

$$\mathrm{R}_{\mathrm{Met}} = \left[1 - \exp\left(\frac{-\mathrm{TR}}{\mathrm{T1}_{\mathrm{Met}}}\right)\right] \cdot \exp\left(\frac{-\mathrm{TE}}{\mathrm{T2}_{\mathrm{Met}}}\right)$$

Full parameters are presented in Supplementary Table 3. Individual tissue fractions were obtained from [19], having been originally derived using the GannetSegment module [20] with segmentation of the corresponding per-subject structural T_1-weighted image performed using the unified tissue segmentation algorithm of SPM12 [21].

Concentration estimates scaled to water but with no adjustment for tissue class (assuming pure water concentration per eq(3) of [22]) were also calculated, hereafter denoted "/H_2O_{noTC}"; see Equation 2.

$$[\mathrm{M}]_{/\mathrm{H_2O_{noTC}}} = \frac{S_\mathrm{M}}{S_\mathrm{H_2O}} \cdot [\mathrm{H_2O}] \cdot Vis_\mathrm{H_2O} \cdot \frac{\#H_\mathrm{H_2O}}{\#H_\mathrm{Met}} \cdot \frac{1-\exp\left(\frac{-\mathrm{TR}}{\mathrm{T1_{H_2O}}}\right)}{1-\exp\left(\frac{-\mathrm{TR}}{\mathrm{T1_{Met}}}\right)} \cdot \frac{\exp\left(\frac{-\mathrm{TE}}{\mathrm{T2_{H_2O}}}\right)}{\exp\left(\frac{-\mathrm{TE}}{\mathrm{T2_{Met}}}\right)} \cdot \frac{MM}{\kappa}$$

Equation 2

Subsequent sections D.1 to D.7 detail the water scaling procedure available within the individual algorithms; scaling factors described therein are reversed before applying Equation 1 and Equation 2 to the raw ratio of intensities.

Parameter	Value	Source and Remarks
S_{M}		Modelled metabolite signal intensity
$f_{TC,vol}$		Tissue volume fractions, for TC of [GM, WM, CSF]
**Water**		
$S_{\mathrm{H_2O}}$		Modelled water signal intensity
$[\mathrm{H_2O}]$	55510 mM	Molar concentration of pure water
$Vis_{\mathrm{H_2O}}$	0.65	Assumed MR-visible water, approximated for pure WM from [23]
β_{GM}	0.78	Tissue-dependent water content for GM [23]
β_{WM}	0.65	Tissue-dependent water content for WM [23]
β_{CSF}	0.97	Tissue-dependent water content for CSF [23]
$\mathrm{T1_{H_2O}}$	1.1 s	Average of WM and GM from [24]
$\mathrm{T1_{H_2O,\,GM}}$	1.331 s	[24]
$\mathrm{T1_{H_2O,\,WM}}$	0.832 s	[24]
$\mathrm{T1_{H_2O,\,CSF}}$	3.817 s	[25]
$\mathrm{T2_{H_2O}}$	0.095 s	Average of WM and GM from [24]
$\mathrm{T2_{H_2O,\,GM}}$	0.11 s	[24]
$\mathrm{T2_{H_2O,\,WM}}$	0.0792 s	[24]
$\mathrm{T2_{H_2O,\,CSF}}$	0.503 s	[26]
$\#H_{\mathrm{H_2O}}$	2	Number of protons contributing to measured water signal
**GABA**		
$\mathrm{T1_{Met}}$	1.31 s	[27]
$\mathrm{T2_{Met}}$	0.088 s	[28]; note that [29] report slightly shorter T1 and T2 for GABA (0.8, 0.13 s respectively).
$\#H_{\mathrm{Met}}$	2	Number of protons contributing to measured GABA signal
MM	0.45	Correction factor for the assumed proportion of macromolecule signal in the measured GABA+ peak [30]; [31] reports 0.46 from simulations. Commonly-assumed value, but note that this factor is expected to be implementation-dependent, based on length and shape of editing pulses (and any applied macromolecule suppression).
κ	0.5	Assumed editing efficiency of GABA [30]; note that [31] reports 0.41 for MEGA-PRESS.
**Glx**		
$\mathrm{T1_{Met}}$	1.23 s	[32]
$\mathrm{T2_{Met}}$	0.18 s	[33]
$\#H_{\mathrm{Met}}$	1	Gannet and AMARES model one proton around 3.75 ppm; basis set methods incorporate signals from four more protons, around 2.15, 2.45 ppm.
MM	1.0	
κ	0.4	Gannet adopts this value, citing FID-A simulation at TE=68ms
**tCr**		No MM/κ factors for tCr (edit-OFF)
$\mathrm{T1_{Met}}$	1.350 s	[34]
$\mathrm{T2_{Met}}$	0.154 s	[34]
$\#H_{\mathrm{Met}}$	5	

Supplementary Table 3 Parameters for water-scaled metabolite estimates

D.1 FSL-MRS

FSL-MRS models the time-domain water reference data with a Voigt decay function [35], then takes the integral of that function's Fourier transform over the 1.65-7.65 ppm range.

Initial molar concentration estimates were obtained assuming 50% GM, 50% WM (i.e., $f_{GM,vol}$ = 0.5 and $f_{WM,vol}$ =0.5). Molar scaling in FSL-MRS is equivalent to Equation 1, but adopting slightly different relaxation parameters as detailed in Supplementary Table 4.

Parameter	Value	Source and Remarks
**Water**		
$T1_{H_2O,\,GM}$	1.5 s	[24,36–40]
$T1_{H_2O,\,WM}$	0.97 s	[24,36–40]
$T1_{H_2O,\,CSF}$	4.47 s	[38]
$T2_{H_2O,\,GM}$	0.088 s	[24,39,41,42]
$T2_{H_2O,\,WM}$	0.073 s	[24,39,41,42]
$T2_{H_2O,\,CSF}$	2.030 s	[43]
**Metabolite**		Metabolite relaxation parameters adopted in FSL-MRS are derived from an average of NAA, Cr and Cho values.
$T1_{Met}$	1.29 s	[34,37,44,45]
$T2_{Met}$	0.194 s	[29,34,44,45(p201),46,47]
MM	1	MM and κ factors not considered in FSL-MRS
κ	1	MM and κ factors not considered in FSL-MRS

Supplementary Table 4 Relaxation parameters used for water scaling in FSL-MRS (all other parameters are per Supplementary Table 3)

D.2 Gannet

Gannet models the water peak as a Lorentzian multiplied by a Gaussian at the same centre frequency, incorporating a linear baseline into the model. The water area is taken as the sum of that optimized function over the 3.8 - 5.6 ppm range (see https://github.com/richardedden/Gannet3.1/blob/master/GannetFit.m#L743)

Gannet can report a number of different water-scaled estimates, with the possibility for tissue-class correction per [48] and normalisation per [20], with integrated masking and segmentation functionality. The present study does not make use of these features, instead basing findings on metab.ConcIU field, which is calculated equivalently to Equation 2, with [H_2O] = 55000 mM and all other parameters per Supplementary Table 3. See also https://github.com/richardedden/Gannet3.1/blob/master/GannetFit.m#L1546

D.3 jMRUI: AMARES

Although jMRUI provides basic water referencing mechanisms internally, based on the amplitude of the reference FID, this functionality could not be batched effectively. Therefore, a separate AMARES run was used to model the water reference, using a superposition of one Gaussian and one Lorentzian component to mimic a pseudo-Voigt model. The centre frequency for the two components was constrained to be equal, with starting values for frequency and linewidth of 4.65 ppm and 7 Hz respectively; zero-order phase and begin time were set to zero.

Intrinsic amplitudes are reported for both the water reference and the metabolite models (i.e., S_M and S_{H2O} terms are available directly), hence there is no need to reverse any applied scaling in this case.

D.4 jMRUI: QUEST

Water referencing is performed identically to the AMARES case (D.3), using the same values for water amplitude.

D.5 LCModel

LCModel measures area of the water peak by integration: it first locates the maximum value of the frequency-domain water reference data within the expected range (4.65 +/- 1 ppm), then integrates the phase-corrected water reference +/-2 ppm around that maximum, assuming a linear baseline between the border regions. Refer LCModel.f lines 546, 4940, 5063 (available via http://www.s-provencher.com/lcmodel.shtml).

LCModel applies a scaling factor to the data, such that the signal strength per proton resonance is consistent between the data and the basis set (see also the LCModel user manual, http://www.s-provencher.com/pub/LCModel/manual/manual.pdf section 10.2):

$$f_{scale} = {Basis_{norm}}/{Water_{norm}}$$

$$Basis_{norm} = {S_{\mathrm{M}}}/{[\#H_{\mathrm{Met}} \cdot Conc_{Met} \cdot ATTMET]}$$

$$Water_{norm} = {S_{\mathrm{H_2O}}}/{[\#H_{\mathrm{H_2O}} \cdot WCONC \cdot ATTH2O]}$$

In this case, metabolite parameters are for NAA, the metabolite specified for basis set scaling (see C.5); $Conc_{Met}$. and $ATTMET$ are appropriate to the basis set (both 1.0), $\#H_{\mathrm{Met}}$ = 3 for the 2.01 ppm NAA peak. Given a consistently-scaled basis set, this is equivalent to scaling of the form:

$$\frac{S_{\mathrm{M}}}{S_{\mathrm{H_2O}}} \cdot \#H_{\mathrm{H_2O}} \cdot WCONC \cdot ATTH2O$$

ATTH2O accounts for the attenuation of NMR-visible water signal due to additional relaxation effects, approximated as $\exp\left(\frac{-\mathrm{TE}}{\mathrm{T2}_{\mathrm{H_2O}}}\right)$. WCONC specifies the NMR-visible water concentration ($[\mathrm{H_2O}] \cdot Vis_{\mathrm{H_2O}}$). Default values of ATTH2O = 0.43 (for TE = 68 ms MEGA-PRESS, see section C.5) and WCONC = 35880 (pure WM) are adopted.

D.6 Osprey

Osprey uses a simulated water basis function to model the water reference, on a fit range from 2-7.4 ppm.

While the standardised processing pipeline evaluates the difference spectrum as diff = (edit-OFF - edit-ON), Osprey assumes a different convention for this: (edit-OFF - edit-ON) / 2. Therefore, an additional factor-of-two correction is needed in this instance.

Raw water scaled estimates (without tissue correction) are obtained. These are calculated similarly to Equation 2, but without macromolecule and edit efficiency terms:

$$\frac{S_{\mathrm{M}}}{S_{\mathrm{H_2O}}} \cdot [\mathrm{H_2O}] \cdot Vis_{\mathrm{H_2O}} \cdot \frac{\#H_{\mathrm{H_2O}}}{\#H_{\mathrm{Met}}} \cdot \frac{1-\exp\left(\frac{-\mathrm{TR}}{\mathrm{T1}_{\mathrm{H_2O}}}\right)}{1-\exp\left(\frac{-\mathrm{TR}}{\mathrm{T1}_{\mathrm{Met}}}\right)} \cdot \frac{\exp\left(\frac{-\mathrm{TE}}{\mathrm{T2}_{\mathrm{H_2O}}}\right)}{\exp\left(\frac{-\mathrm{TE}}{\mathrm{T2}_{\mathrm{Met}}}\right)}$$

In this scaling, Osprey assumes $[\mathrm{H_2O}]$ = 55500 mM, and slightly different parameters for Glx and tCr, including updated $\mathrm{T2}_{\mathrm{Met}}$ estimates from [47]; relaxation estimates in this mode are averaged across tissue class and constituent metabolites (Glu + Gln for Glx, Cr + PCr for tCr). All other parameters are in accordance with Supplementary Table 3.

Parameter	Value	Source and Remarks
**Water**		
$[H_2O]$	55500 mM	
**Glx**		
$T1_{Met}$	1.265 s	[32,34]
$T2_{Met}$	0.133 s	[47]
**Cr**		
$T1_{Met}$	1.350 s	[34]
$T2_{Met}$	0.156 s	[47]

Supplementary Table 5: Relaxation parameters used for water scaling in Osprey (all other parameters per Supplementary Table 3)

See also: https://github.com/schorschinho/osprey/blob/develop/quantify/OspreyQuantify.m#L492

D.7 Tarquin

Tarquin uses the infinity norm (peak absolute amplitude) of the time-domain water reference data for normalisation to water. The reported value is given as:

$$\frac{S_M}{S_{\mathrm{H2O}}} \cdot \#H_{\mathrm{H2O}} \cdot \mathrm{WCONC} \cdot \mathrm{WATT}$$

Where WCONC represents the NMR-visible water concentration (equivalent to $[\mathrm{H_2O}] \cdot Vis_{\mathrm{H_2O}}$), and WATT accounts for the reduction of measured water signal relative to metabolite signal due to differences in T2 relaxation (equivalent to $\frac{\exp\left(\frac{-\mathrm{TE}}{\mathrm{T2_{H_2O}}}\right)}{\exp\left(\frac{-\mathrm{TE}}{\mathrm{T2_{Met}}}\right)}$); in this study, the WCONC and WATT parameters are specified as 35880 mM (pure white-matter value) and 0.76 respectively, noting that the latter is unlikely to be optimal for TE = 68 ms. Internally, S_M is pre-scaled by a factor of 0.5 to account for internal basis-set scaling conventions. Refer: https://github.com/martin3141/tarquin/blob/master/src/common/Workspace.hpp#L469

E Creatine Reference: Edit-OFF sub-spectrum modelling

For modelling of the edit-OFF sub-spectra by basis set algorithms (FSL-MRS, QUEST, LCModel, Osprey and Tarquin), a standard simulated basis set specific to each hardware vendor was adopted. These were derived for edit-OFF sub-spectra using a similar method to that used for the difference spectra (as detailed in section 2.3.1), incorporating a default set of basis components as defined within Osprey: ascorbate (Asc), aspartate (Asp), creatine (Cr), a negative correction term for the Creatine CH_2 singlet around 3.94 ppm (CrCH2), GABA, glycerophosphocholine (GPC), GSH Gln Glu, H_2O, myo-inositol (Ins), lactate (Lac), NAA, NAAG, phosphocholine (PCh), phosphocreatine (PCr), phosphoethanolamine (PE), scyllo-inositol (Scyllo), taurine (Tau), tyrosine (Tyros), and a series of macromolecule and lipid components: $MM_{0.9}$ $MM_{1.2}$ $MM_{1.4}$ $MM_{1.7}$ $MM_{2.0}$ $Lip_{0.9}$ $Lip_{1.3}$ $Lip_{2.0.}$

E.1 FSL-MRS

Edit-OFF sub-spectra were quantified with the standard edit-OFF basis set, using Cr+PCr as an internal reference for basis set scaling. For expedience, the default Newton optimisation algorithm was used; otherwise, all parameters were the same as for the difference case.

```
--basis            ge_off_8192_4000.basis
--data             S12_GABA_68.off.nii.gz
--h2o              S12_GABA_68.ref.nii.gz
--output           S12_GABA_68.off
--overwrite
--TE               68
--TR               2.0
--tissue_frac      .5 .5 0
--verbose
--report
--combine          NAA NAAG
--combine          Glu Gln GSH
--combine          Cr PCr
--combine          GPC PCh

Defaults:
--internal_ref     Cr PCr
--algo Newton
--ppmlim:          (0.2, 4.2)
--baseline_order:  2
--metab_groups:    0
--h2o_scale:       1.0
```

E.2 Gannet

In addition to the GABA+Glx model, Gannet independently fits a Lorentzian model to NAA around 2.01 ± 0.04 ppm, and a dual-Lorentzian model for Choline and Creatine, the latter centred at 3.02 ppm with a fixed 0.18 ppm separation, all from the edit-OFF sub-spectrum. A minor local modification was made to additionally yield water-referenced estimates from the existing Cr and NAA fits (usually only reported for those metabolites contained in the difference spectrum).

E.3 jMRUI: AMARES

For the edit-OFF sub-spectra, a simple model including only three major peaks and residual water was applied: NAA (2.0 ± 0.05 ppm), Cr (3.0 ± 0.05 ppm), Cho (3.2 ± 0.05 ppm), residual water (4.62 ± 0.08 ppm). Soft constraints on linewidth (2-15 Hz for metabolite, 4-25 Hz for water) and relative phase (±10 degrees for metabolites; unconstrained for water). As with the difference case, zeroth- and first-order phase were set to zero.

E.4 jMRUI: QUEST

The standard per-manufacturer edit-OFF basis sets described above were imported in jMRUI text format, manually aligned for NAA @ 2.0 ppm, before assembly into a jMRUI-format metabolite list. Other model parameters were the same as for the difference spectra.

E.5 LCModel

Edit-OFF sub-spectra were modelled with the standard per-manufacturer edit-OFF basis sets. Basic configuration was similar to that used for the difference spectra, with two key exceptions. `PPMGAP` was not defined – which implies fitting over the full defined fit range (0.2 - 4.2 ppm), the default mode of operation. `SPTYPE` was also not defined, again implying a default fit: a regular spline baseline was modelled (with default knot spacing 0.15 ppm), Creatine was used for internal basis set scaling, and LCModel's default set of soft constraints were adopted.

E.6 Osprey

Edit-OFF sub-spectra were modelled using the standard per-manufacturer edit-OFF basis sets, with otherwise similar parameters to those used for the difference spectra. This happened in the same invocation of OspreyFit.

E.7 Tarquin

Edit-OFF sub-spectra were quantified with the standard per-manufacturer edit-OFF basis sets, with HSVD water removal disabled (`--water_width 0`) but all other parameters equivalent to those used for the difference spectra. Tarquin (internal) used `--int_basis 1h_brain`.

```
tarquin

--format      lcm
--input       S12_GABA_68.off.RAW
--input_w     S12_GABA_68.off.REF

--echo        0.068
--fs          5000
--ft          127714400
--auto_phase  false
--auto_ref    false
--water_eddy  false
--water_width 0

--w_att       0.76
--w_conc      35880

--output_txt  S12_GABA_68.off.txt
--output_csv  S12_GABA_68.off.csv
--output_pdf  S12_GABA_68.off.pdf
--output_fit  S12_GABA_68.off.fit
--ext_pdf     true
```

For “Tarquin” (with standard basis set):

```
--basis_lcm   ge_off_4096_5000.basis
```

Or for “Tarquin (internal)” (internal basis set):

```
--int_basis   1h_brain
```

F Quality Control

a) R1: Preprocessing

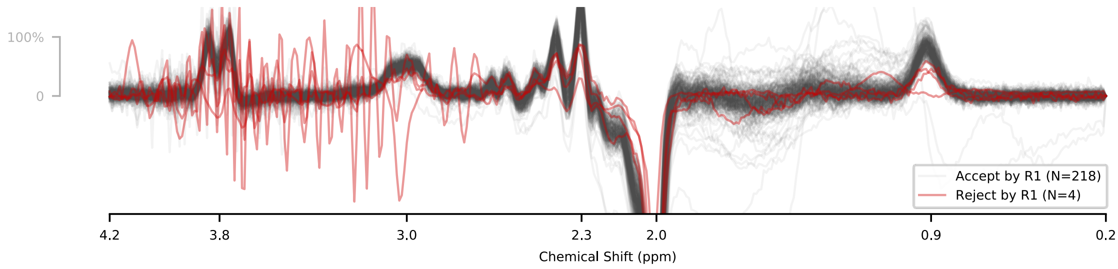

b) R2: SNR, Linewidth (NAA_{diff})

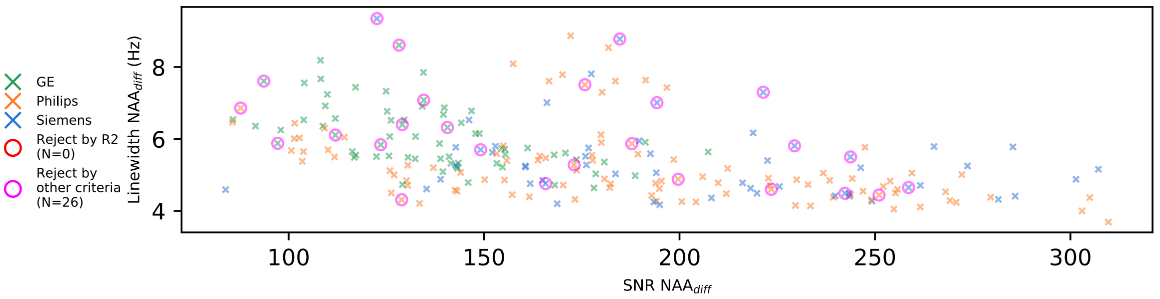

c) R3: Uncertainty (%SD, CRLB or %FitError)

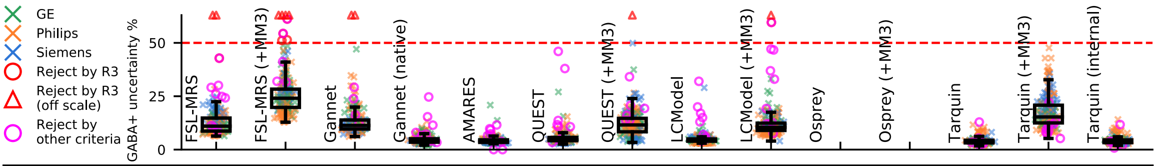

Uncertainty Metric	CRLB	CRLB	FitError	FitError	SD	SD	SD	CRLB	CRLB	N/A	N/A	SD	SD	SD
GABA+ uncertainty %	11±3.8	24±6.4	11±3	4.1±1.1	3.9±0.86	4.8±1.1	12±4.8	4±1.5	11±2.4	-	-	3.8±0.92	15±5.3	3.9±0.93
Glx uncertainty %	12±2.8	12±3.2	6.2±1.6	2.9±0.72	7.6±2.2	4.8±1.3	4.5±1.2	3±1.5	2±0	-	-	1.4±0.4	1.4±0.39	3.3±1.2
tCr uncertainty %	8.7±2.1	8.7±2.1	2.1±0.3	1.4±0.19	2.2±0.66	6.4±2.9	6.4±2.9	1±0	1±0	-	-	0.8±0.33	0.8±0.33	1±0.47
Reject by R3	3	8	2	0	0	0	1	0	2	0	0	0	0	0

d) R4: Median Absolute Deviation (MAD) from global median

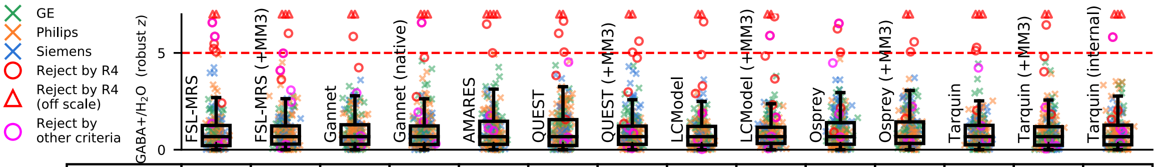

Reject by R4	9	7	4	6	6	7	8	7	8	5	5	6	6	6
Invalid/Failed	0	0	1	1	0	0	0	0	0	0	0	2	2	1
TOTAL	11	11	7	9	9	10	11	9	10	8	8	9	10	9
Total GE	2 (2%)	3 (4%)	2 (2%)	4 (5%)	2 (2%)	3 (4%)	4 (5%)	2 (2%)	2 (2%)	2 (2%)	4 (5%)	3 (4%)	2 (2%)	2 (2%)
Total Philips	2 (1%)	1 (0%)	3 (2%)	2 (1%)	1 (0%)	3 (2%)	3 (2%)	3 (2%)	3 (2%)	2 (1%)	1 (0%)	2 (1%)	4 (3%)	2 (1%)
Total Siemens	7 (12%)	7 (12%)	2 (3%)	3 (5%)	6 (11%)	4 (7%)	4 (7%)	4 (7%)	5 (9%)	4 (7%)	3 (5%)	4 (7%)	4 (7%)	5 (9%)

Supplementary Figure 6: Quality control; rejected fits for each criterion, according to algorithm. A single dataset may be flagged by multiple rejection criteria.

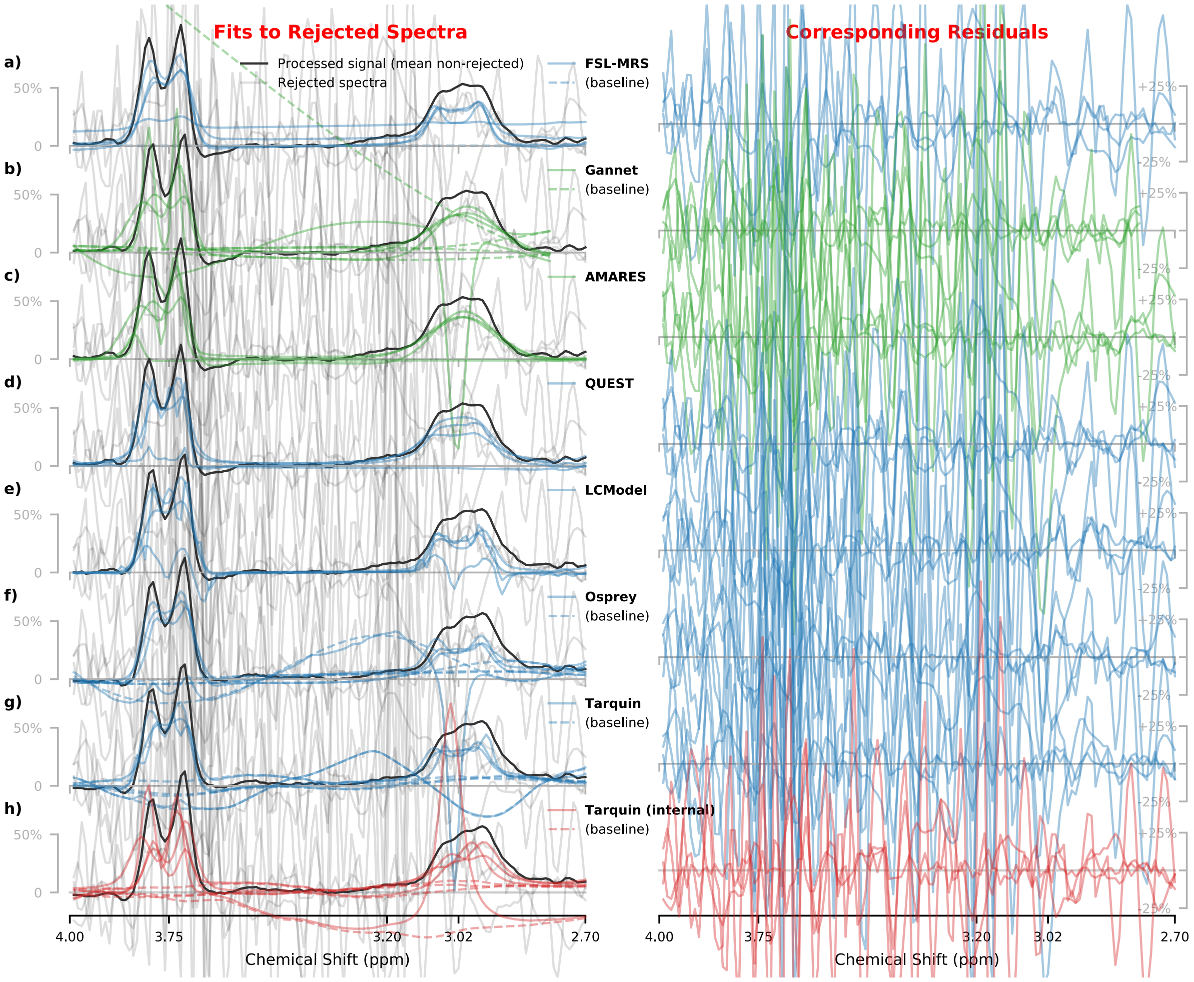

Supplementary Figure 7: Most algorithms dutifully applied their model even when supplied with very poor input data, often returning visually pleasing fits which were acceptable according to other criteria.

G Fitting Outcomes per algorithm

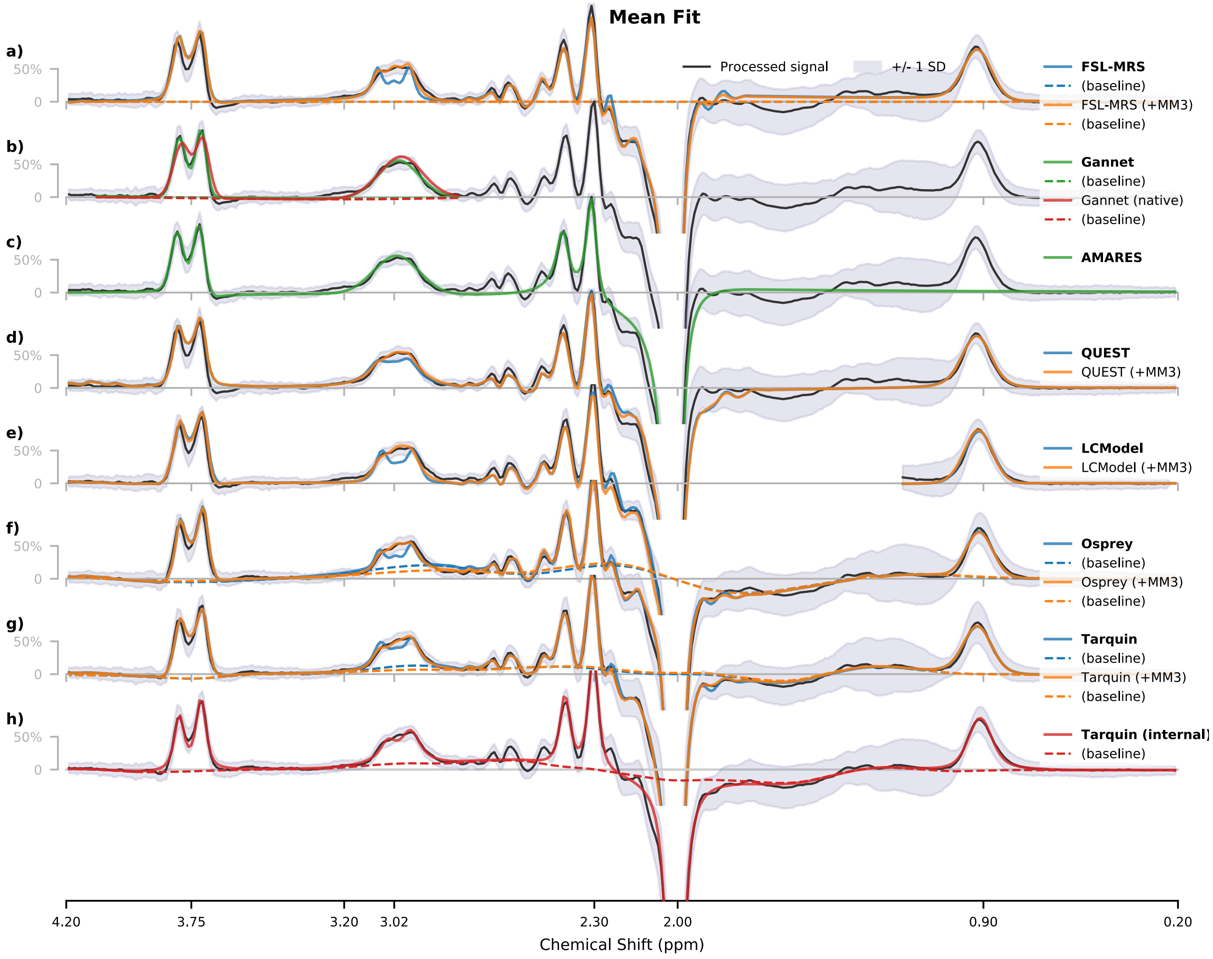

Supplementary Figure 8: Average metabolite and baseline (where applicable) models with corresponding residuals for the GABA+ edited spectra, for each algorithm. Vertical scaling is normalised. This represents the same data as Figure 2, on the full fit range for each algorithm

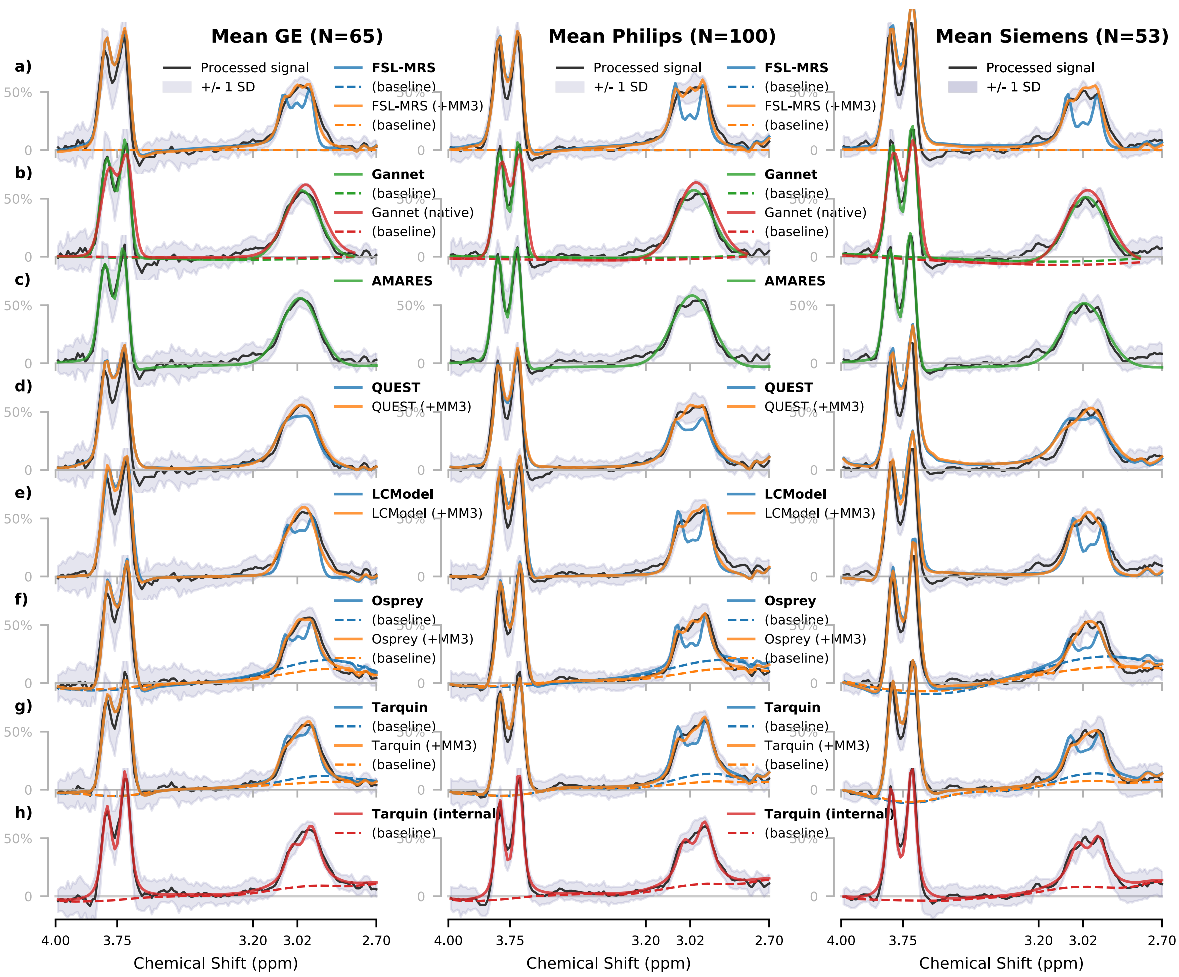

Supplementary Figure 9: Average metabolite and baseline (where applicable) models for the GABA+ edited spectra, for each algorithm and each vendor. Vertical scaling is normalised. This represents the same data as Figure 2, split according to vendor.

H Agreement between algorithms

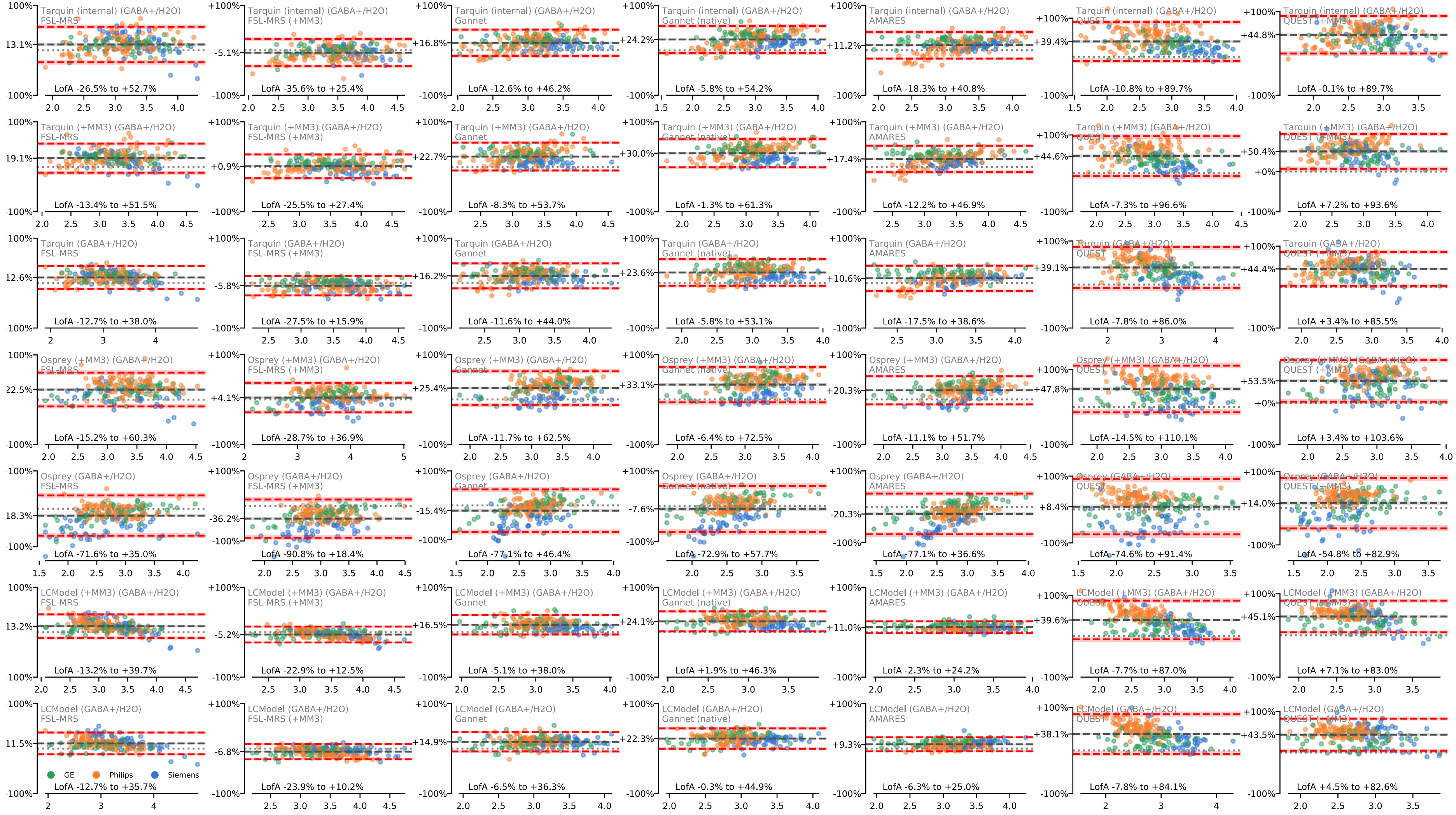

Supplementary Figure 10: Bland-Altman plots, comparing pairs of estimates for GABA+/ H_2O and GABA+/tCr (continued overleaf…)

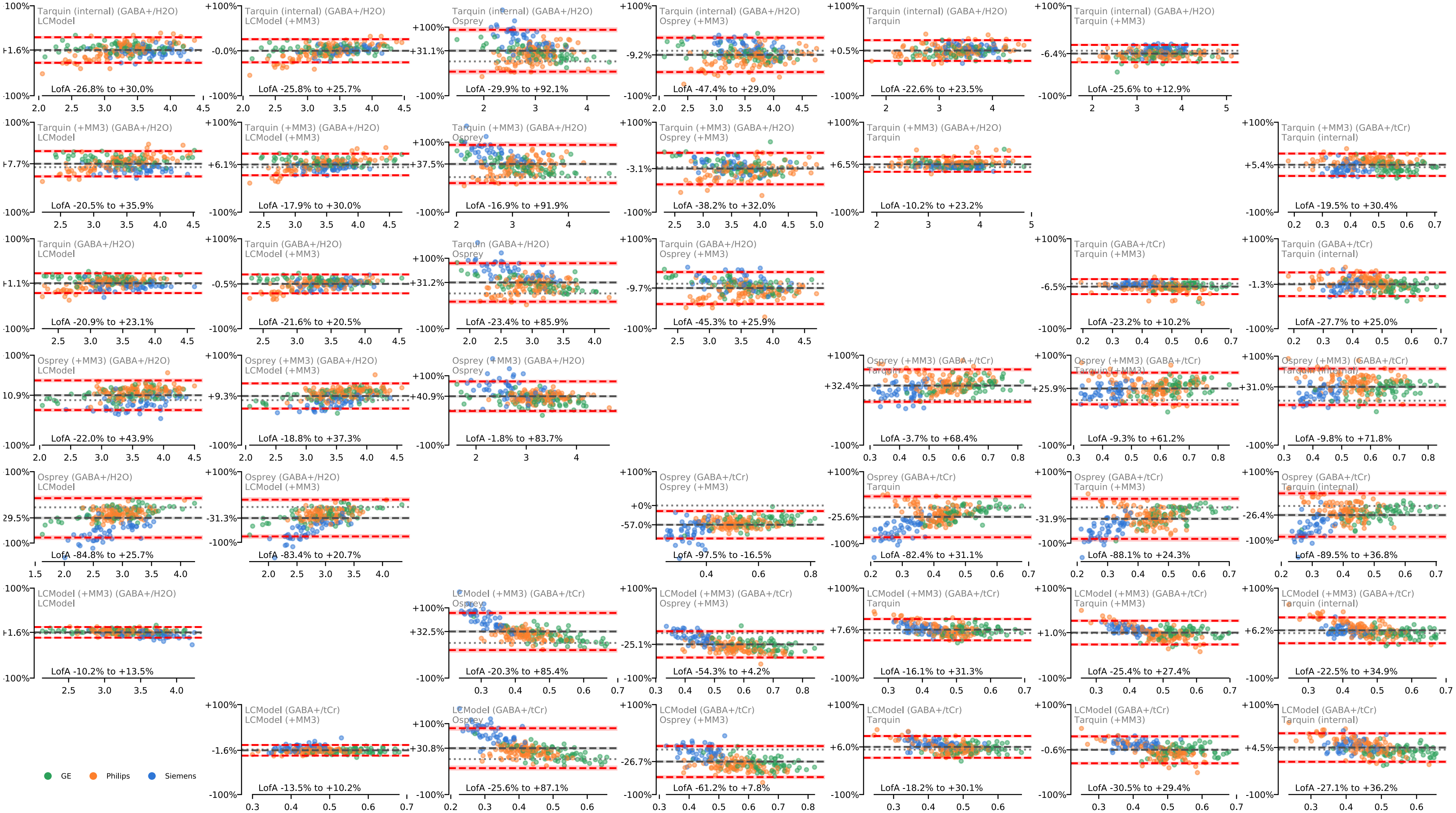

Supplementary Figure 10 (…continued…)

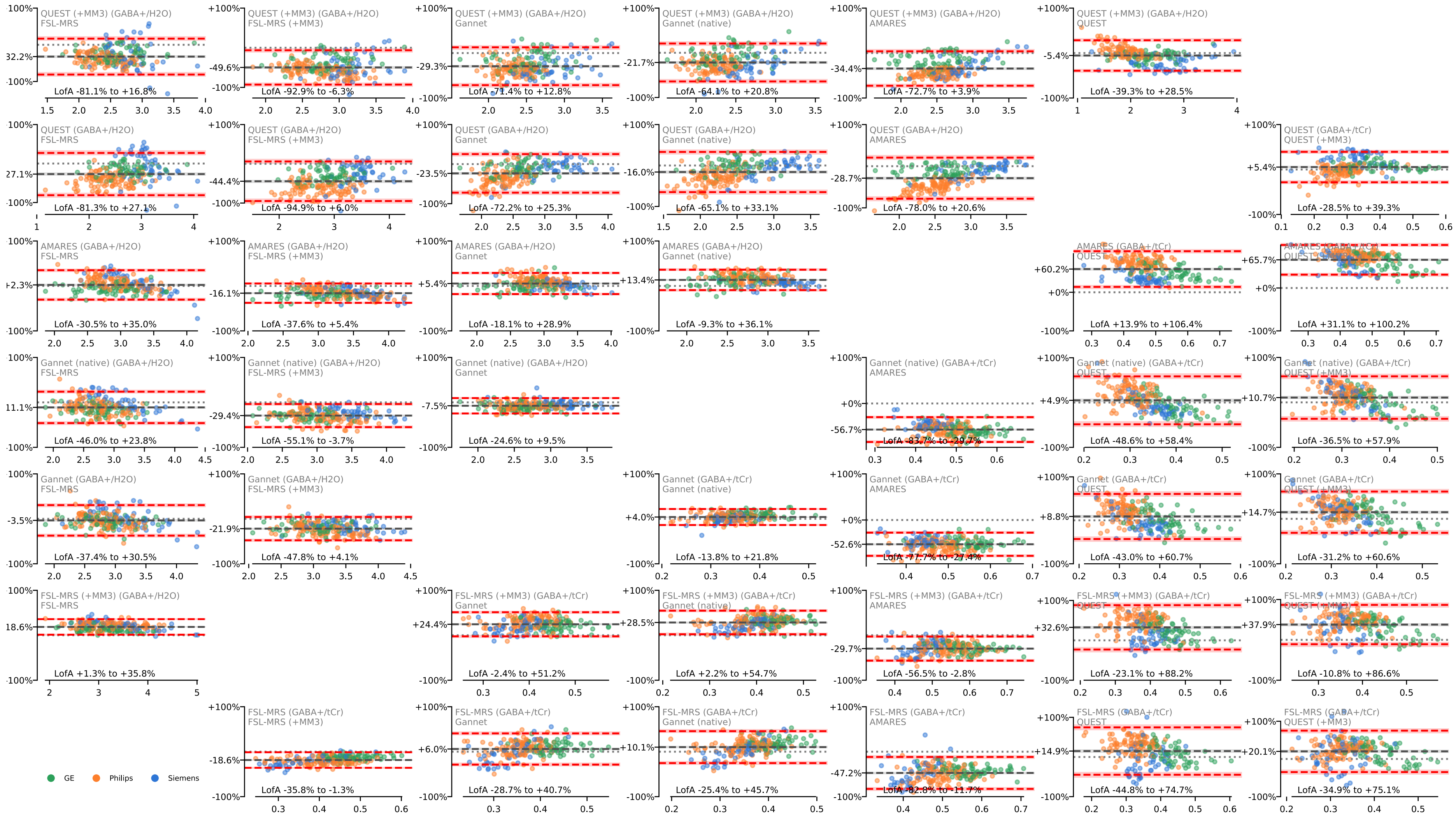

Supplementary Figure 10 (…continued…)

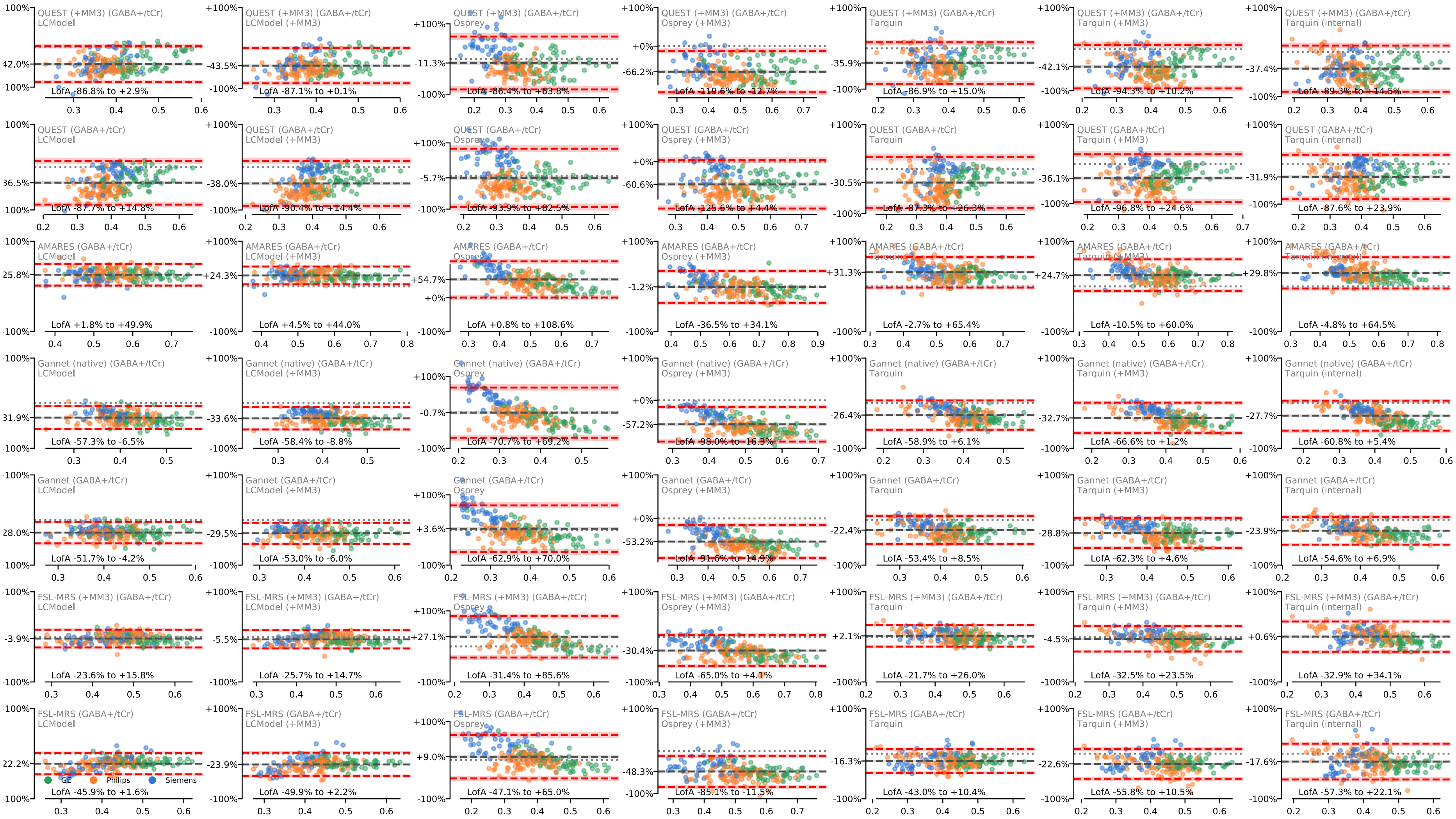

Supplementary Figure 10 (…continued)

Supplementary Figure 11: Distribution of metabolite estimates obtained from each algorithm, grouped by vendor

a)

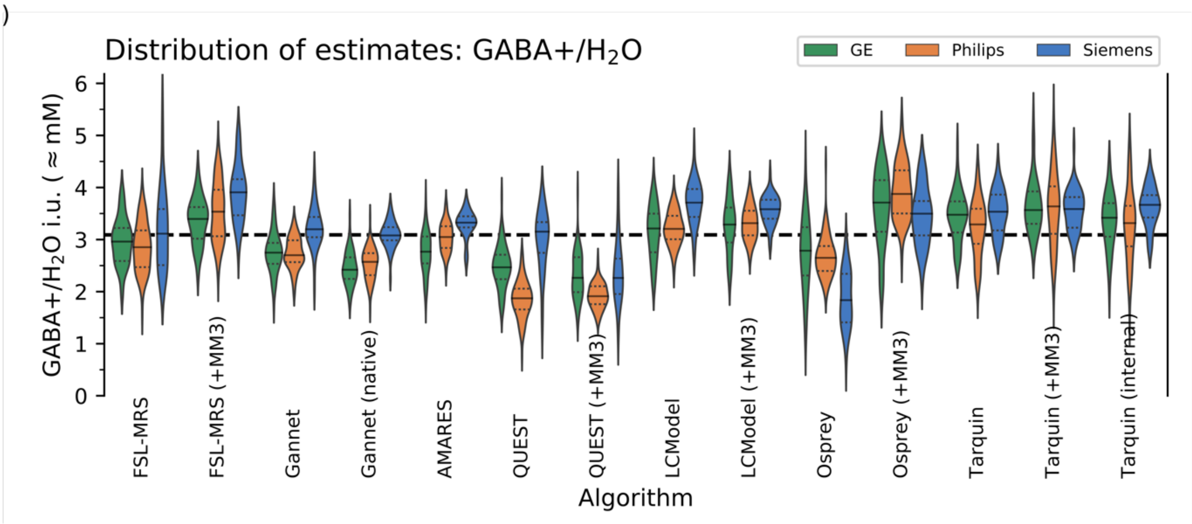

b)

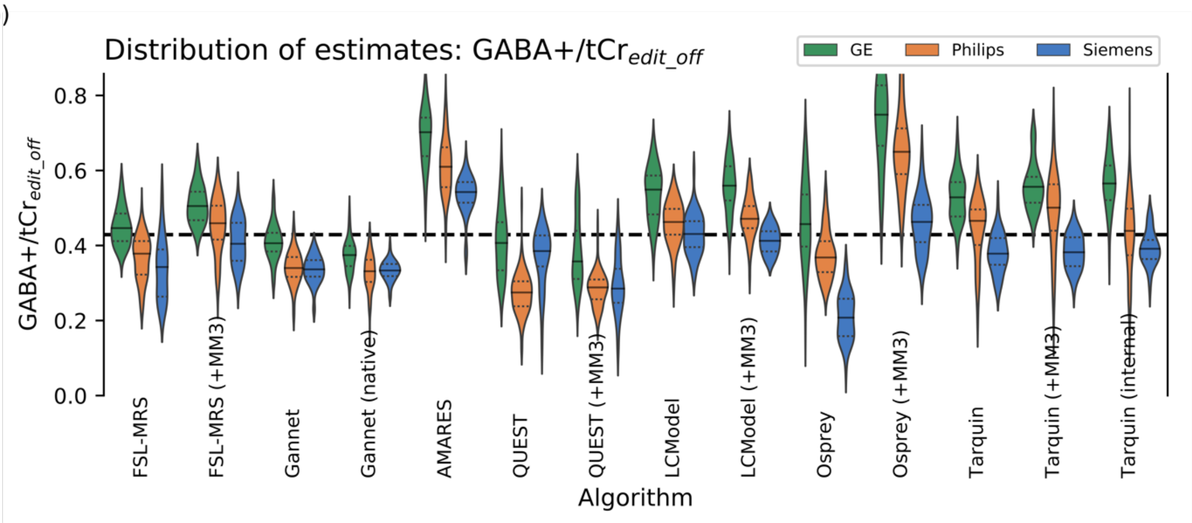

c)

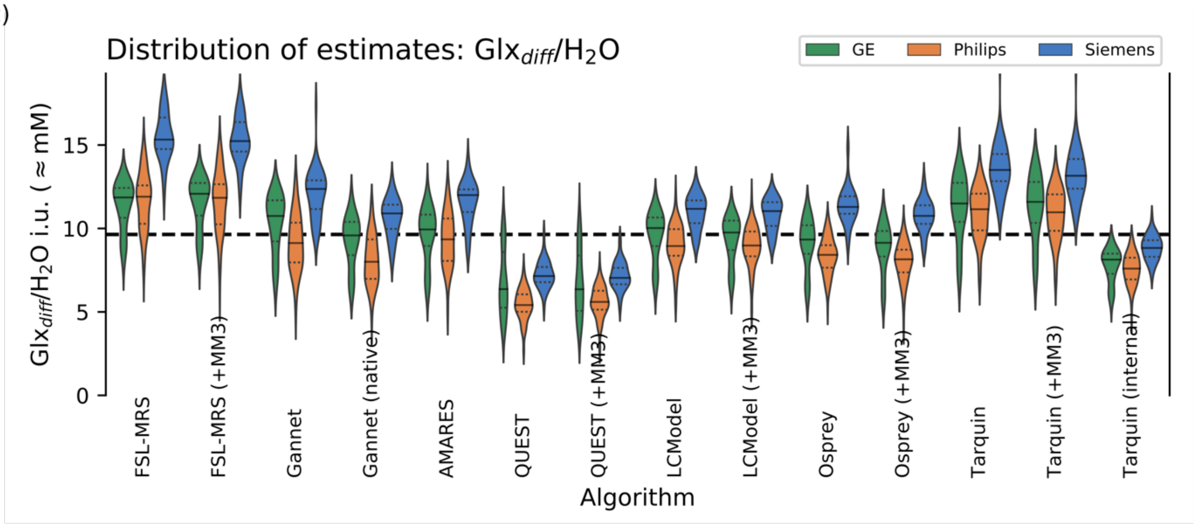

d)

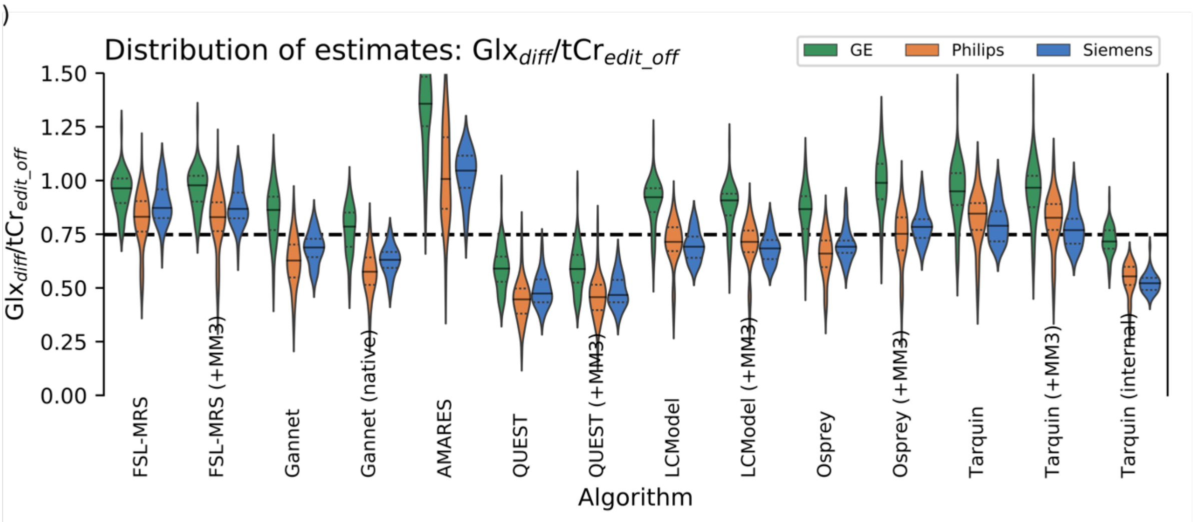
Distribution of estimates: Glx_diff/tCr_edit_off
GE
Philips
Siemens
Glx_diff/tCr_edit_off
1.50
1.25
1.00
0.75
0.50
0.25
0.00
FSL-MRS
FSL-MRS (+MM3)
Gannet
Gannet (native)
AMARES
QUEST
QUEST (+MM3)
LCModel
LCModel (+MM3)
Osprey
Osprey (+MM3)
Tarquin
Tarquin (+MM3)
Tarquin (internal)
Algorithm

e)

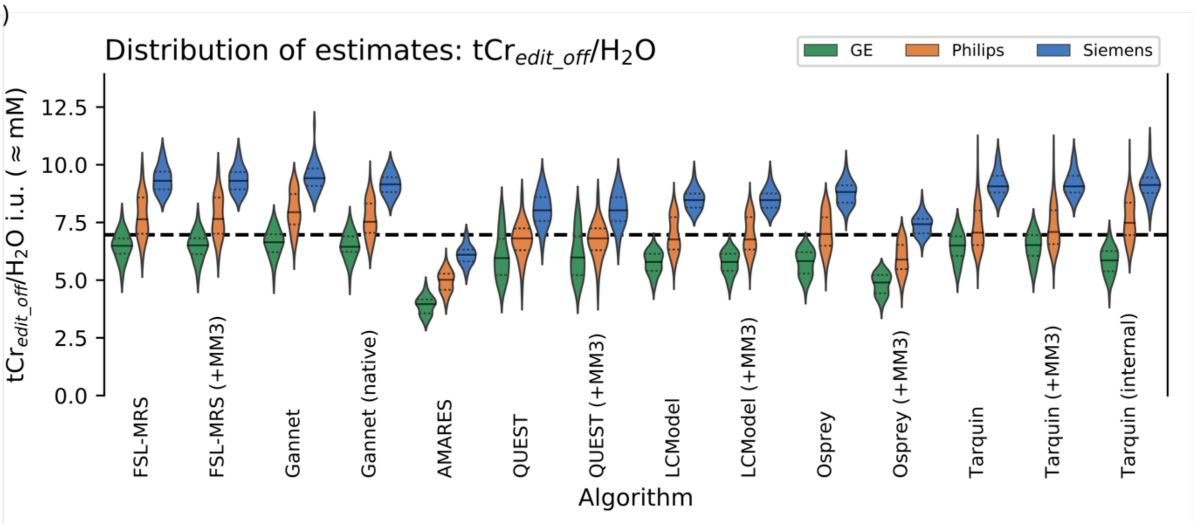
Distribution of estimates: tCr_edit_off/H2O
GE
Philips
Siemens
tCr_edit_off/H2O i.u. (≈ mM)
12.5
10.0
7.5
5.0
2.5
0.0
FSL-MRS
FSL-MRS (+MM3)
Gannet
Gannet (native)
AMARES
QUEST
QUEST (+MM3)
LCModel
LCModel (+MM3)
Osprey
Osprey (+MM3)
Tarquin
Tarquin (+MM3)
Tarquin (internal)
Algorithm

	FSL-MRS	FSL-MRS (+MM3)	Gannet	Gannet (native)	AMARES	QUEST	QUEST (+MM3)	LCModel	LCModel (+MM3)	Osprey	Osprey (+MM3)	Tarquin	Tarquin (+MM3)	Tarquin (internal)	Median
GABA+/H2O mean ±SD	2.950 ±0.567	3.541 ±0.559	2.837 ±0.409	2.644 ±0.388	3.059 ±0.375	2.230 ±0.633	2.072 ±0.456	3.308 ±0.462	3.379 ±0.396	2.538 ±0.639	3.707 ±0.620	3.441 ±0.509	3.591 ±0.588	3.448 ±0.593	3.190 ±0.401
GABA+/H2O diff GE	+0.4%	-4.1%	-3.1%	* -8.5%	*** -9.5%	+10.6%	+9.3%	-2.9%	-2.7%	+9.8%	+0.1%	+1.0%	-0.7%	-0.9%	-1.4%
GABA+/H2O diff Philips	-3.3%	-0.2%	-4.8%	-2.7%	-0.3%	*** -16.1%	*** -7.7%	-3.1%	-1.9%	+4.4%	+4.6%	-4.4%	+1.3%	-3.8%	-2.9%
GABA+/H2O diff Siemens	+5.6%	* +10.4%	*** +12.7%	*** +16.5%	*** +8.7%	*** +41.3%	+9.2%	*** +12.1%	** +6.0%	*** -27.6%	-5.7%	+2.7%	-0.1%	+6.3%	** +5.8%
GABA+/tCr mean ±SD	0.393 ±0.076	0.466 ±0.072	0.358 ±0.055	0.341 ±0.046	0.611 ±0.092	0.321 ±0.094	0.301 ±0.081	0.477 ±0.074	0.471 ±0.077	0.366 ±0.123	0.639 ±0.146	0.466 ±0.090	0.497 ±0.104	0.458 ±0.106	0.441 ±0.076
GABA+/tCr diff GE	*** +13.6%	*** +8.4%	*** +13.5%	*** +9.8%	*** +14.9%	*** +26.5%	*** +18.9%	*** +15.1%	*** +18.9%	*** +24.9%	*** +17.1%	*** +13.5%	*** +11.9%	*** +23.4%	*** +17.3%
GABA+/tCr diff Philips	-3.7%	-1.5%	-5.0%	-2.9%	-0.2%	*** -14.5%	** -4.1%	-3.0%	+0.1%	+0.6%	+1.6%	+0.0%	+0.7%	-4.2%	-1.1%
GABA+/tCr diff Siemens	-12.8%	*** -13.2%	* -6.0%	-2.3%	*** -11.2%	+19.8%	-5.2%	*** -9.7%	*** -12.3%	*** -43.2%	*** -27.6%	*** -18.8%	*** -23.2%	*** -14.6%	*** -14.3%
Glx/H2O mean ±SD	12.299 ±2.289	12.410 ±2.251	10.352 ±2.009	9.256 ±1.749	9.980 ±1.781	6.167 ±1.526	6.279 ±1.473	9.868 ±1.464	9.663 ±1.414	9.244 ±1.715	8.881 ±1.633	11.783 ±1.978	11.683 ±1.914	8.124 ±1.039	9.847 ±1.610
Glx/H2O diff GE	** -3.6%	-2.7%	+3.8%	+3.5%	-0.4%	+3.2%	+1.2%	+1.6%	+1.0%	+1.0%	+2.9%	-2.4%	-0.8%	+0.1%	+1.2%
Glx/H2O diff Philips	-3.2%	-4.6%	*** -11.8%	*** -13.5%	-6.3%	*** -12.3%	*** -10.8%	* -9.3%	* -7.1%	*** -8.9%	*** -8.2%	* -5.4%	-6.1%	-6.5%	** -10.1%
Glx/H2O diff Siemens	*** +24.6%	*** +22.8%	*** +19.5%	*** +17.8%	*** +20.2%	*** +15.9%	*** +12.1%	*** +13.3%	*** +14.2%	*** +22.1%	*** +21.1%	*** +14.6%	*** +12.6%	*** +8.7%	*** +15.7%
Glx/tCr mean ±SD	0.883 ±0.123	0.880 ±0.130	0.698 ±0.145	0.640 ±0.127	1.102 ±0.236	0.485 ±0.113	0.491 ±0.110	0.746 ±0.134	0.737 ±0.129	0.699 ±0.133	0.805 ±0.161	0.859 ±0.139	0.850 ±0.141	0.575 ±0.107	0.749 ±0.134
Glx/tCr diff GE	*** +9.2%	*** +11.2%	*** +23.7%	*** +22.8%	*** +23.2%	*** +21.8%	*** +19.8%	*** +23.6%	*** +23.2%	*** +24.0%	*** +22.9%	*** +10.6%	*** +13.7%	*** +24.7%	*** +21.9%
Glx/tCr diff Philips	* -5.8%	* -5.7%	*** -10.0%	*** -10.0%	-8.6%	** -7.8%	* -6.9%	* -4.3%	-3.1%	*** -5.6%	*** -6.6%	-1.5%	-2.8%	*** -3.6%	* -5.0%
Glx/tCr diff Siemens	-1.2%	-1.3%	-1.3%	-1.4%	** -5.1%	-2.2%	-4.8%	*** -7.3%	*** -7.2%	-1.1%	-2.6%	-8.1%	** -9.5%	*** -9.1%	* -6.7%
tCr/H2O mean ±SD	7.368 ±1.287	7.370 ±1.276	7.794 ±1.267	7.496 ±1.175	4.814 ±0.897	6.838 ±1.114	6.845 ±1.114	6.652 ±1.189	6.634 ±1.185	6.719 ±1.291	5.694 ±1.089	7.098 ±1.281	7.109 ±1.278	7.275 ±1.412	7.072 ±1.223
tCr/H2O diff GE	*** -12.0%	*** -11.7%	*** -14.8%	*** -14.0%	*** -17.7%	*** -12.9%	*** -12.6%	*** -13.0%	*** -12.8%	*** -13.3%	*** -14.0%	*** -8.4%	*** -8.3%	*** -19.5%	*** -13.9%
tCr/H2O diff Philips	+3.7%	+3.8%	+1.9%	+0.4%	+4.2%	-0.4%	-0.5%	+1.7%	+2.0%	+4.2%	+3.5%	-0.6%	-0.3%	+2.9%	+1.1%
tCr/H2O diff Siemens	*** +26.3%	*** +26.2%	*** +20.8%	*** +22.1%	*** +26.7%	*** +17.4%	*** +17.3%	*** +27.3%	*** +27.6%	*** +31.3%	*** +30.4%	*** +27.7%	*** +27.5%	*** +25.4%	*** +24.4%

*Supplementary Table 6: Mean concentration estimates (institutional units, ≈ mM) from each algorithm, reported across all subjects. Estimates grouped by vendor are expressed as a % difference relative to the mean across all subjects for the respective algorithm; significance indicated by *, **, *** for p_{holm} < .05, .01 and .001 respectively.*

I Additional Correlation Analyses

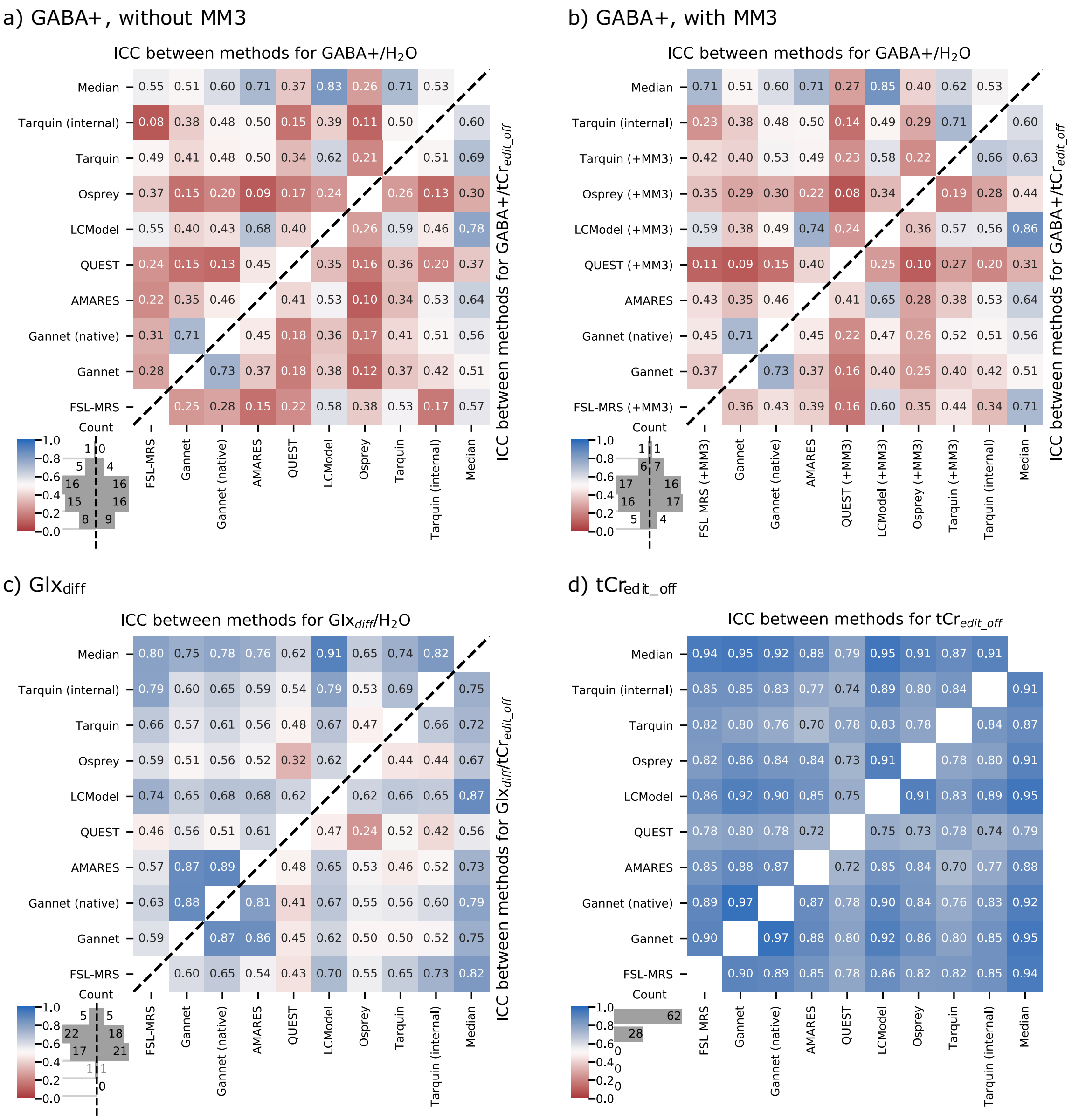

Supplementary Figure 12 Intra-class correlation between algorithms, for additional metabolites and ratios

I.1 Glx$_{diff}$, Glx$_{edit_off}$

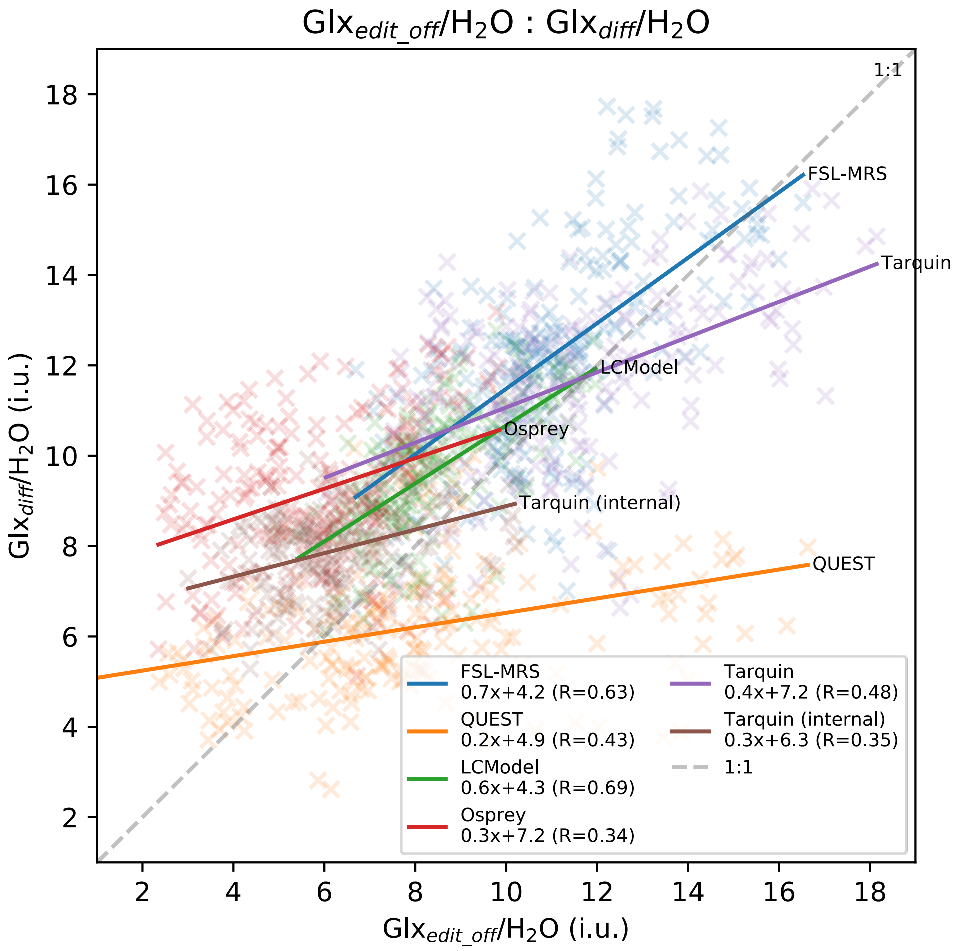

Supplementary Figure 13: Estimates for Glx$_{edit_off}$ vs Glx$_{diff}$ for each algorithm which modelled both.

J Supplementary References

1. Murdoch JB, Dydak U. Modeling MEGA-PRESS macromolecules for a better grasp of GABA. In: ; 2011:1394. https://cds.ismrm.org/protected/11MProceedings/files/1394.pdf

2. Moser P, Hingerl L, Strasser B, et al. Whole-slice mapping of GABA and GABA+ at 7T via adiabatic MEGA-editing, real-time instability correction, and concentric circle readout. *NeuroImage*. 2019;184:475-489. doi:10.1016/j.neuroimage.2018.09.039

3. Považan M, Hnilicová P, Hangel G, et al. Detection of MM using metabolite-nulled MEGA-LASER at 3T–A possible effect on GABA+ signal. In: *Proc Intl Soc Mag Reson Med*. Vol 25. ; 2017:1058.

4. Clarke WT, Stagg CJ, Jbabdi S. FSL-MRS: An end-to-end spectroscopy analysis package. *Magn Reson Med*. 2021;85(6):2950-2964. doi:10.1002/mrm.28630

5. Edden RAE, Puts NAJ, Harris AD, Barker PB, Evans CJ. Gannet: A batch-processing tool for the quantitative analysis of gamma-aminobutyric acid-edited MR spectroscopy spectra: Gannet: GABA Analysis Toolkit. *J Magn Reson Imaging*. 2014;40(6):1445-1452. doi:10.1002/jmri.24478

6. Naressi A, Couturier C, Devos JM, et al. Java-based graphical user interface for the MRUI quantitation package. *Magma Magn Reson Mater Phys Biol Med*. 2001;12(2-3):141-152. doi:10.1007/BF02668096

7. Stefan D, Cesare FD, Andrasescu A, et al. Quantitation of magnetic resonance spectroscopy signals: the jMRUI software package. *Meas Sci Technol*. 2009;20(10):104035. doi:10.1088/0957-0233/20/10/104035

8. Vanhamme L, van den Boogaart A, Van Huffel S. Improved Method for Accurate and Efficient Quantification of MRS Data with Use of Prior Knowledge. *J Magn Reson*. 1997;129(1):35-43. doi:10.1006/jmre.1997.1244

9. Graveron-Demilly D. Quantification in magnetic resonance spectroscopy based on semi-parametric approaches. *Magn Reson Mater Phys Biol Med*. 2014;27(2):113-130. doi:10.1007/s10334-013-0393-4

10. Ratiney H, Coenradie Y, Cavassila S, van Ormondt D, Graveron-Demilly D. Time-domain quantitation of 1 H short echo-time signals: background accommodation. *MAGMA Magn Reson Mater Phys Biol Med*. 2004;16(6):284-296. doi:10.1007/s10334-004-0037-9

11. Provencher SW. Estimation of metabolite concentrations from localizedin vivo proton NMR spectra. *Magn Reson Med*. 1993;30(6):672-679. doi:10.1002/mrm.1910300604

12. Oeltzschner G, Zöllner HJ, Hui SCN, et al. Osprey: Open-source processing, reconstruction & estimation of magnetic resonance spectroscopy data. *J Neurosci Methods*. 2020;343:108827. doi:10.1016/j.jneumeth.2020.108827

13. Reynolds G, Wilson M, Peet A, Arvanitis TN. An algorithm for the automated quantitation of metabolites in in vitro NMR signals. *Magn Reson Med*. 2006;56(6):1211-1219. doi:10.1002/mrm.21081

14. Wilson M, Reynolds G, Kauppinen RA, Arvanitis TN, Peet AC. A constrained least-squares approach to the automated quantitation of in vivo ^{1} H magnetic resonance spectroscopy data: Automated Quantitation of In Vivo ^{1} H MRS Data. *Magn Reson Med*. 2011;65(1):1-12. doi:10.1002/mrm.22579

15. Levitt MH. *Spin Dynamics: Basics of Nuclear Magnetic Resonance*. John Wiley & Sons; 2001.

16. Smith SA, Levante TO, Meier BH, Ernst RR. Computer Simulations in Magnetic Resonance. An Object-Oriented Programming Approach. *J Magn Reson A*. 1994;106(1):75-105. doi:10.1006/jmra.1994.1008

17. Govindaraju V, Young K, Maudsley AA. Proton NMR chemical shifts and coupling constants for brain metabolites. *NMR Biomed*. 2000;13(3):129-153. doi:10.1002/1099-1492(200005)13:3<129::aid-nbm619>3.0.co;2-v

18. Gasparovic C, Chen H, Mullins PG. Errors in ^{1} H-MRS estimates of brain metabolite concentrations caused by failing to take into account tissue-specific signal relaxation. *NMR Biomed*. 2018;31(6):e3914. doi:10.1002/nbm.3914

19. Mikkelsen M, Rimbault DL, Barker PB, et al. Big GABA II: Water-referenced edited MR spectroscopy at 25 research sites. *NeuroImage*. 2019;191:537-548. doi:10.1016/j.neuroimage.2019.02.059

20. Harris AD, Puts NAJ, Edden RAE. Tissue correction for GABA-edited MRS: Considerations of voxel composition, tissue segmentation, and tissue relaxations: Tissue Correction for GABA-Edited MRS. *J Magn Reson Imaging*. 2015;42(5):1431-1440. doi:10.1002/jmri.24903

21. Ashburner J, Friston KJ. Unified segmentation. *NeuroImage*. 2005;26(3):839-851. doi:10.1016/j.neuroimage.2005.02.018

22. Rideaux R, Mikkelsen M, Edden RAE. Comparison of methods for spectral alignment and signal modelling of GABA-edited MR spectroscopy data. *NeuroImage*. 2021;232:117900. doi:10.1016/j.neuroimage.2021.117900

23. Ernst T, Kreis R, Ross BD. Absolute Quantitation of Water and Metabolites in the Human Brain. I. Compartments and Water. *J Magn Reson B*. 1993;102(1):1-8. doi:10.1006/jmrb.1993.1055

24. Wansapura JP, Holland SK, Dunn RS, Ball WS. NMR relaxation times in the human brain at 3.0 tesla. *J Magn Reson Imaging JMRI*. 1999;9(4):531-538. doi:10.1002/(sici)1522-2586(199904)9:4<531::aid-jmri4>3.0.co;2-l

25. Lu H, Nagae-Poetscher LM, Golay X, Lin D, Pomper M, van Zijl PCM. Routine clinical brain MRI sequences for use at 3.0 Tesla. *J Magn Reson Imaging*. 2005;22(1):13-22. doi:10.1002/jmri.20356

26. Piechnik SK, Evans J, Bary LH, Wise RG, Jezzard P. Functional changes in CSF volume estimated using measurement of water T_2 relaxation. *Magn Reson Med*. 2009;61(3):579-586. doi:10.1002/mrm.21897

27. Puts NAJ, Barker PB, Edden RAE. Measuring the longitudinal relaxation time of GABA in vivo at 3 tesla. *J Magn Reson Imaging*. 2013;37(4):999-1003. doi:10.1002/jmri.23817

28. Edden RAE, Intrapiromkul J, Zhu H, Cheng Y, Barker PB. Measuring T2 in vivo with J-difference editing: Application to GABA at 3 tesla. *J Magn Reson Imaging*. 2012;35(1):229-234. doi:10.1002/jmri.22865

29. Träber F, Block W, Lamerichs R, Gieseke J, Schild HH. ^{1}H metabolite relaxation times at 3.0 tesla: Measurements of T1 and T2 values in normal brain and determination of regional differences in transverse relaxation: ^{1}H Metabolite Relaxation Times at 3.0 T. *J Magn Reson Imaging*. 2004;19(5):537-545. doi:10.1002/jmri.20053

30. Mullins PG, McGonigle DJ, O'Gorman RL, et al. Current practice in the use of MEGA-PRESS spectroscopy for the detection of GABA. *NeuroImage*. 2014;86:43-52. doi:10.1016/j.neuroimage.2012.12.004

31. Near J, Simpson R, Cowen P, Jezzard P. Efficient γ-aminobutyric acid editing at 3T without macromolecule contamination: MEGA-SPECIAL: γ-AMINOBUTYRIC ACID EDITING USING MEGA-SPECIAL. *NMR Biomed*. 2011;24(10):1277-1285. doi:10.1002/nbm.1688

32. Posse S, Otazo R, Caprihan A, et al. Proton echo-planar spectroscopic imaging ofJ-coupled resonances in human brain at 3 and 4 Tesla. *Magn Reson Med*. 2007;58(2):236-244. doi:10.1002/mrm.21287

33. Ganji SK, Banerjee A, Patel AM, et al. T_2 measurement of J-coupled metabolites in the human brain at 3T: T_2 OF J-COUPLED METABOLITES AT 3T. *NMR Biomed*. 2012;25(4):523-529. doi:10.1002/nbm.1767

34. Mlynárik V, Gruber S, Moser E. Proton T_1 and T_2 relaxation times of human brain metabolites at 3 Tesla: METABOLITE T_1 AND T_2 IN HUMAN BRAIN AT 3 T. *NMR Biomed*. 2001;14(5):325-331. doi:10.1002/nbm.713

35. Whittenburg SL. Bayesian analysis of time-domain Voigt functions. *Spectrochim Acta A Mol Biomol Spectrosc*. 1996;52(10):1169-1174. doi:10.1016/0584-8539(96)01685-6

36. Dieringer MA, Deimling M, Santoro D, et al. Rapid Parametric Mapping of the Longitudinal Relaxation Time T1 Using Two-Dimensional Variable Flip Angle Magnetic Resonance Imaging at 1.5 Tesla, 3 Tesla, and 7 Tesla. Zhan W, ed. *PLoS ONE*. 2014;9(3):e91318. doi:10.1371/journal.pone.0091318

37. Ethofer T, Mader I, Seeger U, et al. Comparison of longitudinal metabolite relaxation times in different regions of the human brain at 1.5 and 3 Tesla. *Magn Reson Med*. 2003;50(6):1296-1301. doi:10.1002/mrm.10640

38. Rooney WD, Johnson G, Li X, et al. Magnetic field and tissue dependencies of human brain longitudinal1H2O relaxation in vivo. *Magn Reson Med*. 2007;57(2):308-318. doi:10.1002/mrm.21122

39. Stanisz GJ, Odrobina EE, Pun J, et al. T1, T2 relaxation and magnetization transfer in tissue at 3T. *Magn Reson Med*. 2005;54(3):507-512. doi:10.1002/mrm.20605

40. Wright PJ, Mougin OE, Totman JJ, et al. Water proton T 1 measurements in brain tissue at 7, 3, and 1.5T using IR-EPI, IR-TSE, and MPRAGE: results and optimization. *Magn Reson Mater Phys Biol Med*. 2008;21(1-2):121-130. doi:10.1007/s10334-008-0104-8

41. Bartha R, Michaeli S, Merkle H, et al. In vivo $^{1}H_2O$ T_2^* measurement in the human occipital lobe at 4T and 7T by Carr-Purcell MRI: Detection of microscopic susceptibility contrast. *Magn Reson Med*. 2002;47(4):742-750. doi:10.1002/mrm.10112

42. Gelman N, Gorell JM, Barker PB, et al. MR Imaging of Human Brain at 3.0 T: Preliminary Report on Transverse Relaxation Rates and Relation to Estimated Iron Content. *Radiology*. 1999;210(3):759-767. doi:10.1148/radiology.210.3.r99fe41759

43. Spijkerman JM, Petersen ET, Hendrikse J, Luijten P, Zwanenburg JJM. T 2 mapping of cerebrospinal fluid: 3 T versus 7 T. *Magn Reson Mater Phys Biol Med*. 2018;31(3):415-424. doi:10.1007/s10334-017-0659-3

44. An L, Li S, Shen J. Simultaneous determination of metabolite concentrations, T_1 and T_2 relaxation times: Metabolite T_1 and T_2 at 7 T. *Magn Reson Med*. 2017;78(6):2072-2081. doi:10.1002/mrm.26612

45. Li Y. T1 and T2 Metabolite Relaxation Times in Normal Brain at 3T and 7T. *J Mol Imaging Dyn*. 2013;02(02). doi:10.4172/2155-9937.S1-002

46. Marjańska M, Auerbach EJ, Valabrègue R, Van de Moortele PF, Adriany G, Garwood M. Localized ^{1}H NMR spectroscopy in different regions of human brain *in vivo* at 7 T: T_2 relaxation times and concentrations of cerebral metabolites: REGIONAL DIFFERENCES IN T_2 AND METABOLITE CONCENTRATIONS AT 7 T. *NMR Biomed*. 2012;25(2):332-339. doi:10.1002/nbm.1754

47. Wyss PO, Bianchini C, Scheidegger M, et al. In vivo estimation of transverse relaxation time constant (T_2) of 17 human brain metabolites at 3T: T_2 of 17 Human Brain Metabolites at 3T. *Magn Reson Med*. 2018;80(2):452-461. doi:10.1002/mrm.27067

48. Gasparovic C, Song T, Devier D, et al. Use of tissue water as a concentration reference for proton spectroscopic imaging. *Magn Reson Med*. 2006;55(6):1219-1226. doi:10.1002/mrm.20901

K Figures Captions

Figure 1: Processing (b) and modelling (c) workflow, summarising key differences between the algorithms assessed.

Figure 2 Average metabolite and baseline (where applicable) models with corresponding residuals for the GABA+ edited spectra, for each algorithm. Vertical scaling is normalised; outcomes over the full fit range are presented in Supplementary Figure 8; outcomes split by vendor are presented in Supplementary Figure 9.

Figure 3: Distribution of GABA+/H_2O estimates from each algorithm, grouped by manufacturer. Global median is shown in dashed black.

Figure 4 Relationship between GABA+ and grey matter, with different modelling strategies for GABA+. Robust (skipped) correlation coefficients are reported, with line-of-best-fit in dashed black.

Figure 5 Intraclass correlation coefficients between algorithms, scaled to water (upper left triangle) and tCr_{edit_off} (lower right triangle), with basis set algorithms excluding (a) and including (b) a component representing co-edited macromolecule contribution. “Median” data denotes correlation with the median estimate across all algorithms.

Supplementary Figure 1: Average metabolite and baseline (where applicable) models with corresponding residuals for each algorithm, baseline model and constraint model in the exploratory analysis. Corresponding fits over the full range are presented in Supplementary Figure 2.

Supplementary Figure 2: Average metabolite and baseline (where applicable) models for each algorithm, baseline model and constraint model in the exploratory analysis.

Supplementary Figure 3: Distribution of metabolite estimates for GABA+ (a), GABA (b), and MM3co (c) obtained from each modelling strategy, grouped by vendor

Supplementary Figure 4: Relationship between GABA+ and grey matter, with different baseline and soft constraint parameters for the MM3 component. Robust (skipped) correlation coefficients are reported, with line-of-best-fit in dashed black

Supplementary Figure 5: Relationship between GABA and grey matter, with different baseline and soft constraint parameters for the MM3 component. Robust (skipped) correlation coefficients are reported, with line-of-best-fit in dashed black

Supplementary Figure 6: Quality control; rejected fits for each criterion, according to algorithm. A single dataset may be flagged by multiple rejection criteria.

Supplementary Figure 7: Most algorithms dutifully applied their model even when supplied with very poor input data, often returning visually pleasing fits which were acceptable according to other criteria.

Supplementary Figure 8: Average metabolite and baseline (where applicable) models with corresponding residuals for the GABA+ edited spectra, for each algorithm. Vertical scaling is normalised. This represents the same data as Figure 2, on the full fit range for each algorithm

Supplementary Figure 9: Average metabolite and baseline (where applicable) models for the GABA+ edited spectra, for each algorithm and each vendor. Vertical scaling is normalised. This represents the same data as Figure 2, split according to vendor.

Supplementary Figure 10: Bland-Altman plots, comparing pairs of estimates for GABA+/ H_2O and GABA+/tCr (continued overleaf…)

Supplementary Figure 11: Distribution of metabolite estimates obtained from each algorithm, grouped by vendor

Supplementary Figure 12 Intra-class correlation between algorithms, for additional metabolites and ratios

Supplementary Figure 13: Estimates for Glx_{edit_off} vs Glx_{diff} for each algorithm which modelled both.

Supplementary Table 1: Basic demographics, hardware and software parameters for the constituent datasets

Supplementary Table 2: Mean concentration estimates (institutional units, ≈ mM), for each algorithm in the exploratory analysis. Estimates grouped by vendor are expressed as a % difference relative to the mean across all subjects for the respective algorithm; significance indicated by *, **, *** for $p_{holm} < .05$, .01 and .001 respectively

Supplementary Table 3 Parameters for water-scaled metabolite estimates

Supplementary Table 4 Relaxation parameters used for water scaling in FSL-MRS (all other parameters are per Supplementary Table 3)

Supplementary Table 5: Relaxation parameters used for water scaling in Osprey (all other parameters per Supplementary Table 3)

Supplementary Table 6: Mean concentration estimates (institutional units, ≈ mM) from each algorithm, reported across all subjects. Estimates grouped by vendor are expressed as a % difference relative to the mean across all subjects for the respective algorithm; significance indicated by *, **, *** for $p_{holm} < .05$, .01 and .001 respectively.
